# The Transgenerational Impact of High-Fat Diet and Diabetic Pregnancy on Embryonic Transcriptomics and Mitochondrial Health

**DOI:** 10.3390/biomedicines13082019

**Published:** 2025-08-19

**Authors:** Abigail K. Klein, Benjamin P. Derenge, Malini Mukherjee, Srikrishna P. Reddy, Tricia D. Larsen, Prathapan Ayyappan, Tyler C. T. Gandy, Kyle M. Siemers, Michael S. Kareta, Michelle L. Baack

**Affiliations:** 1 Sanford Research, 2301 E. 60th St. North, Sioux Falls, SD 57104, USAprathapan.ayyappan@sanfordhealth.org (P.A.); michael.kareta@sanfordhealth.org (M.S.K.); michelle.baack@sanfordhealth.org (M.L.B.); 2 Sanford School of Medicine, University of South Dakota, 1400 W. 22nd St., Sioux Falls, SD 57015, USA

**Keywords:** overnutrition, high fat, diabetes, embryo, transcriptome, mitochondria, oxidative stress, transgenerational

## Abstract

**Background/Objectives:** Overnutrition increases comorbidities such as gestational diabetes during pregnancy that can have detrimental consequences for both parent and progeny. We previously reported that high-fat (HF) diet and late-gestation diabetes (DM) incite mitochondrial dysfunction, oxidative stress, and cardiometabolic disease in first generation (F1) rat offspring, partially through epigenomic and transcriptomic programming. Primordial germ cells, which become the second generation (F2), are also exposed, which could incite generational risk. This study aimed to determine whether the F2 transcriptome already has genomic variation at the preimplantation embryo stage, and whether variations normalize, persist or compound in the third generation (F3). **Methods:** F0 female rats were fed a control or HF diet, then DM was induced in HF-fed dams on gestational day (GD)14, exposing F1 offspring and F2 primordial germ cells to hyperlipidemia, hyperglycemia and fetal hyperinsulinemia during the last third of pregnancy. F1 pups were reared by healthy dams and bred to produce F2 embryos (F2e) and F2 pups. F2 offspring were bred to produce F3 embryos (F3e). Embryos were assessed by a novel grading method, live cell imaging, and single-cell RNA sequencing. **Results:** Embryo grades were not different, but HF+DM F2e had more cells while F3e had fewer cells and overall fewer embryos. HF+DM F2e had similar mitochondria quantity but a downregulation of genes involved in lipid metabolism and more oxidative stress, consistent with mitochondrial dysfunction. They also had an upregulation of chromatin-remodeling genes. The predicted developmental effect is accelerated embryo aging and epigenetic drift. In contrast, HF+DM F3e had an adaptive stress response leading to increased mitochondria quantity and an upregulation of genes involved in mitochondrial respiration, metabolism, and genomic repair that led to a predicted developmental effect of delayed embryo maturation. **Conclusions:** Although pathways vary, both generations have metabolically linked differentially expressed genes that influence cell fate and developmental pathways. In conclusion, HF+DM pregnancy can program the early embryonic transcriptome for three generations, despite an intergenerational healthy diet.

## 1. Introduction

Overnutrition is common, affecting 2/3 of reproductive-age women, and it plays a detrimental role in pregnancy and offspring health [1,2,3]. Specifically, diets high in fat contribute to overnutrition and an obese and pro-inflammatory phenotype leading to mitochondrial dysfunction in the ovary and oocyte [3,4,5,6]. Normal expansion of mitochondria during oocyte maturation is crucial for placentation and early embryonic growth but this can be disrupted by dysfunctional mitochondria and subsequent oxidative stress [7]. Both mitochondrial quality and quantity are critical and contribute uniquely to early embryonic growth and differentiation. After fertilization, the first cell-fate decision occurs around the blastocyst stage, resulting in two distinct cell types, the inner cell mass (ICM) and the trophectoderm (TE). TE cells, which give rise to placental trophoblasts, have more mitochondria and produce more ATP to enable high energy processes like implantation and placentation [8]. Disrupting metabolism during early placentation and embryogenesis can increase the risk of placental dysfunction, altered fuel transport, growth abnormalities, and birth defects [9,10,11,12,13,14,15]. It can also introduce epigenetic marks that program susceptibility to long-term metabolic health consequences for offspring, a phenomenon known as developmental programming [16].

Maternal overnutrition also increases the risk for developing gestational diabetes [17], a comorbidity which leads to a combination of hyperglycemia, hyperlipidemia and responsive fetal hyperinsulinemia during the last trimester of pregnancy [18]. Together, this adverse metabolic milieu incites additional metabolic disturbances that further impact fetal growth, development and the long-term health of first-generation (F1) offspring [19,20,21]. Furthermore, because the timing of gestational diabetes coincides with fetal gametogenesis, the primordial germ cells are exposed to the same adverse conditions, which can introduce novel developmental programming for the second generation (F2) [22]. Indeed, mounting evidence demonstrates that overnutrition not only programs metabolic disease susceptibility for F1 offspring, but also for F2 generations, which may be regulated epigenetically [23,24]. While multigenerational programming from overnutrition has been studied extensively in animal models [25,26,27,28,29], there is a remaining gap in knowledge about the effects of maternal overnutrition plus late-gestation diabetes on the embryonic transcriptome and mitochondrial health in second (F2) and third generations (F3). Specifically, there is a need to know whether differentially expressed genes (DEG) persist or are compounded in unexposed F3, rather than returning to normal under the influence of a healthy lifestyle. Understanding generational programing similarities and differences at the earliest stage of development is crucial for decreasing the growing burden of metabolic disease in high-risk populations in the future.

Our lab has extensively characterized a rat model of maternal high-fat diet and late-gestation diabetes which exposes the developing offspring to hyperglycemia, hyperlipidemia and fetal hyperinsulinemia in the last third of pregnancy [30,31,32,33,34,35,36,37]. We followed F1 cardiometabolic health from birth to 12 months and found that like humans, newborn F1 offspring had poorer cardiac function at birth which improved by weaning, but cardiometabolic disease reappeared with aging [30,31,32,33,34,35,36,37,38]. Disease susceptibility was due to fuel-mediated alterations in the myocardial epigenome [33], transcriptome [34] and underlying mitochondrial dysfunction including impaired bioenergetics, unbalanced dynamics (fission > fusion), oxidative stress, and increased mitochondria-mediated cell-death [30,32,36,37,38]. Specifically, high-fat diet mediated an increase in gene-activating marks (H3Ac and H3K4me3) in promoter regions of 28 candidate genes, 15 of which were related to metabolic processes with others related to stress response processes [33]. Cardiac transcriptomic analysis of F1 newborn offspring hearts revealed upregulation of peroxisome proliferator-activated receptor gamma coactivator alpha (PGC1α), which regulates mitochondrial biogenesis, and downregulation of the PI3K/Akt pathway that regulates cell fate [34,37]. Although histone modifications are frequently reversible, H3K4me3 can have stable, multigenerational effects and may also serve as a marker for other permanent gene-suppressive DNA methylation [39].

This study tested whether genomic disruptions from maternal overnutrition and diabetic pregnancy would incite similar or compounded transcriptomic changes that influence metabolism and cell fate in F2 and F3 generation preimplantation embryos, rather than being reversed when the initial environmental stressor is no longer present. We hypothesized that F2 generation embryos (F2e) would have transcriptional perturbations that impact metabolic health and that some, but not all of these transcriptional changes would be mitigated in the F3 generation embryos (F3e) due to the lack of direct exposure.

## 2. Materials and Methods

### 2.1. Animal Care and Ethics

Guidelines from the Animal Welfare Act and the National Institutes of Health Guide for the Care and Use of Laboratory Animals were followed for this study which was under the approval from the Sanford Research Institutional Animal Care and Use Committee (IACUC 170-06-23B and 203-06-26B), and Sanford Research is an AAALAC certified facility. Because generational studies using human embryos are not feasible or ethical, we used a rat model. The Sprague Dawley strain was utilized in this study to build upon our previous extensive characterization of F0 and F1 offspring using this model [30,31,32,33,34,35,36,37,38,40]. We have previously validated drug dosing including streptozotocin and insulin sliding scales for this strain. Sprague Dawley rats are exceptional mothers and are amenable to cross-fostering that was required to decrease lactational confounding. Additionally, it is an outbred model, and inbreeding negatively influences mitochondrial function in high-fat diet-fed mice [41]. Rats were purchased from Inotiv (Indianapolis, IN, USA). Animals had free access to food and water and were housed in a temperature-controlled facility with a 14:10 light–dark cycle.

### 2.2. Diet Characteristics

Dams (7–9 weeks old) were fed either a control diet (CD) or a high-fat (HF) diet for 28 days prior to breeding. The CD was 3.2 kcal/g, providing an estimated daily caloric intake of 16.6% fat, 22.9% protein and 60.5% carbohydrates (TD.170868, Envigo Teklad Diets, Madison, WI, USA). The HF diet was 4.3 kcal/g, providing an estimated daily caloric intake of 39.7% fat, 18.8% protein and 41.4% carbohydrates (TD.95217, Envigo Teklad Diets, Madison, WI, USA). Table S1 highlights differences in the dietary make-up including the source of fat and individual fatty acid composition.

### 2.3. Timed Breeding

Dams at least 10 weeks of age were bred with CD-fed sires. For single cell RNA sequencing (scRNA-seq) experiments, a breeding strategy for sire breeders was used across generations as detailed in Figure S1. Vaginal swabs were taken each morning, then rolled onto a microscope slide to check for presence of spermatozoa which indicated embryonic day (E)0.5 because breeding occurs during dark cycles. Breeding pairs were then separated, and dams were singly housed throughout pregnancy.

### 2.4. Late-Gestation Diabetes Induction

Diabetes was induced on gestational day (GD)14, exposing the late-gestation fetus to hyperglycemia. The timing of diabetes induction was purposefully in the last third of pregnancy, late in gestation, similar to gestational diabetes that is diagnosed between 24 and 28 weeks of gestation in humans [42]. Importantly, this timepoint is after F1 offspring organogenesis, but during F2 primordial germ cell development. On GD14, dams were lightly anesthetized with a mixture of isoflurane–oxygen during which timed pregnancies were confirmed by ultrasonography, then dams were administered either citrate-buffered saline diluent (20 mM, pH 4.5, Thermo Fisher Scientific, Waltham, MA, USA) or 65 mg/kg streptozotocin (STZ) (Millipore-Sigma, Burlington, MA, USA) reconstituted with diluent. Administration was by intraperitoneal injection. Thereafter, tail-nick whole blood glucose levels were monitored. Dams that were injected with STZ but did not have a non-fasting blood glucose level ≥ 200 mg/dL were excluded from the study. This strategy resulted in two groups of F0 dams, CD-fed, non-diabetic controls (CON) and HF-fed, diabetic dams (HF+DM). Dams in the HF+DM group were treated twice daily with sliding-scale insulin using regular insulin in the morning (Humalin Regular U100, Eli Lilly, Indianapolis, IN, USA) and longer acting insulin-glargine in the evening (Lantus U-100, Eli Lilly, Indianapolis, IN, USA) to maintain a range of 200–400 mg/dL of non-fasting, whole blood glucose levels, similar to pre-treatment range as previously detailed [30,32,33,34,37].

### 2.5. Generational Animal Model

**Figure 1:**
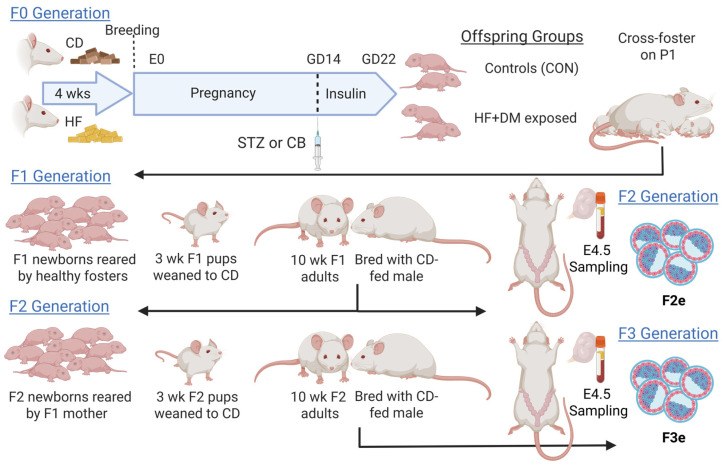
Generational experimental animal model. F0 dams were fed a control (CD) or high-fat (HF) diet for 4 weeks before breeding with CD-fed males. At gestational day (GD)14, HF-fed dams were injected with streptozotocin (STZ) to induce diabetes (DM) and CD-fed dams were injected with sham citrate buffer (CB). DM dams were treated twice daily with sliding-scale insulin. Dams delivered naturally to produce F1 control (CON) and high fat with diabetes (HF+DM) exposed pups, which were cross-fostered to CD-fed dams and weaned to CD, then later used to generate F2 pups and F2 embryos (F2e). F2 pups were fed CD and grown to generate F3 embryos (F3e). Blood, ovaries and embryos were collected on E4.5 from F2 and F3 offspring dams. E = embryonic day, GD = gestational day, P = postnatal day, wk = week. Created in BioRender. Klein, Abigail. (2025) https://BioRender.com/q3nxb80 [43].

Experimental methods for generational studies are outlined in Figure 1. Specifically, F0 dams were allowed to deliver pups naturally on GD22. On postnatal day (P)1, F1 offspring from both groups were culled to 8 pups/litter and cross-fostered to a timed-pregnant, CD-fed, non-diabetic dam that had also delivered within one week. Cross fostering consistently occurred at 2–4 h after the start of the light cycle to minimize differences attributed to lactation or stress during rearing by HF+DM F0 dams that received twice daily handling for insulin injections. Because growth restriction or macrosomia can impact offspring health and developmental programming, birthweights were considered when stratifying F1 and F2 pups for generational studies. In our hands, Sprague Dawley rats have a wide range of birthweights from 2.38 to 9.46 g, but most are between 5.74 (the 25th percentile) and 6.22 (the 75th percentile). When possible, we selected experimental F1 and F2 females with birthweights between the 25th–75th percentile. At 3 weeks of age, F1 pups were weaned to CD. At 10 weeks of age, F1 females were bred with unexposed, CD-fed males yielding F2e on E4.5 and F2 pups. F2 pups were also fed CD and grown to 10 weeks of age, then bred with unexposed males to produce F3e. Embryos used for scRNA-seq experiments were generated using a limited male breeder method shown in Figure S1.

### 2.6. Body Weights and Blood Sampling

At baseline, post diet (after 4 weeks of diet) and the time of harvest (either E4.5 or P1), body weights were recorded and whole blood was collected through tail nick or jugular vein draws. Tail nicks were used for testing glucose levels using a glucometer (Precision Xtra Blood Glucose & Ketone Monitoring System, Abbott Laboratories, Abbott Park, IL, USA). Dams were lightly anesthetized using a mixture of isofluorane–oxygen for jugular vein draws in which a 23-guage sterile needle with 3 mL syringe was used to collect whole blood using an aseptic technique. Blood was transferred from syringe to EDTA tube and kept on ice until processing. A whole blood aliquot was taken then the remaining sample was centrifuged for 5 min at 5000 rpm to separate cells and plasma. Whole blood and plasma were stored at −80 °C until analysis. Plasma triglyceride levels were quantified using a commercially available kit (Point Scientific Triglyceride Reagent Kit, Fisher Scientific, Lenexa, KS, USA) with colorimetric detection according to the manufacturer’s instruction.

### 2.7. Embryo Collection and Culture

On E4.5, dams were anesthetized using a mixture of isoflurane–oxygen, then whole blood was collected by pericardial puncture. Euthanasia was confirmed by removal of the heart. The ovaries and uterine horns were trimmed of fat and the intact horns were placed into pre-warmed 1 × DPBS (Thermo Fisher Scientific, Waltham, MA, USA). Preimplantation embryos were flushed out of each uterine horn using a 16-guage needle and identified using the EVOS FL fluorescent cell imaging system (Advanced Microscopy Group, Thermo Fisher Scientific, Waltham, MA, USA) at 4× magnification with bright field detection, then placed into a pre-warmed drop of embryo media overlaid with oil (OVOIL^TM^, Vitrolife, Denver, CO, USA). EmbryoLove^TM^ embryo media (EmbryoTech^TM^ Laboratories Inc., Haverhill, MA, USA) was used for all embryo experiments, and embryos were handled using the Stripper^TM^ Micropipettor (CooperSurgical^®^, Inc., Trumbull, CT, USA). Fresh embryos were cultured for 2–4 h at 37 °C in 5% CO_2_ to obtain similarly mature blastocysts before embryo grading, staining and scRNA-seq. The number of embryos used for each experiment is detailed in Table S2.

### 2.8. Embryo Grade and Viability

Embryos were graded following a 2–4 h culture period. Bright field images were taken before and after culture using the EVOS system as described above. Post-culture images were graded using objective, pre-defined criteria as detailed below. While the Gardner grading system for human embryos has been extensively described and utilized in assistive reproductive technology [44], there is not a similar ubiquitous method for rat embryos. Moreover, Gardner grading uses a combination of numerical and letter values that are not conducive to group comparisons. Therefore, we developed a rat-specific embryo grading system that converts classical embryo grades to a numerical score (continuous variable) using Gardner principles, which includes expansion classification, inner cell mass (ICM) quality, and trophectoderm (TE) quality [45]. Our grading weighs ICM quality slightly higher since it is the strongest predictor of live birth [46]. Table 1 shows the details of the pre-defined criteria for grading rat E4.5 embryos.

To convert the descriptive grades of the embryos (4AA, 3AB, 1CC, etc.) into numerical scores, the following equation was used:

$$Embryo\;Score(ES)=EXS+ICMS\left\{\begin{array}{c} A=3\\ B=2\\ C=1 \end{array}+TS\left\{\begin{array}{c} A\;or\;B=2\\ C=1 \end{array}\right.\right.$$

To minimize subjectivity and bias, scoring methods were consistently applied by a single reviewer after the scoring process was validated. Validation occurred by two blinded reviewers that each scored 113 embryos with high agreement as determined by a high inter-rater reliability with a weighted Kappa of 0.766.

Embryo viability was also assessed using pre- and post-culture embryo grading. We defined the rate of non-viable embryos as the number of 1CC grade embryos that did not grow or mature in culture (had no changes in morphology or cell count) divided by the total number of embryos in the litter.

### 2.9. Embryo Staining and Imaging

Following the 2–4 h embryo culture, a subset of embryos were stained and imaged to evaluate mitochondria and oxidative stress. Embryos selected for staining were at the proper E4.5 developmental stage (mature blastocyst) with a discernable blastocoel and distinct inner cell mass and trophectoderm which correlated to an embryonic grade of greater than or equal to a 3BB. This was carried out to compare similar stage embryos and mitigate confounding variation in staining from dying embryos or those that have arrested at earlier stages of development. After selection, embryos were transferred to one of two different staining solutions: (1) embryo media with Hoechst (1:1000), Mitotracker^TM^ Green FM (1:1000) and CellROX^TM^ Deep Red Reagent (1:1000) or (2) embryo media with Hoechst (1:1000) and BODIPY^TM^ 581/591 C11 (1:1000) for 20 min at 37 °C with 5% CO_2_. All stains were purchased from Thermo Fisher Scientific (Waltham, MA, USA). After incubation, embryos were transferred to a FluroDish^TM^ (World Precision Instruments, Sarasota, FL, USA) with fresh, pre-warmed embryo media overlaid with oil. Embryos stained with Mitotracker^TM^ and CellROX^TM^ were immediately imaged, whereas embryos stained with BODIPY^TM^ C11 were incubated for an additional 40 min prior to imaging. Images were acquired using a Nikon A1R confocal microscope system with a live cell incubation system set at 37 °C with 5% CO_2_ and NIS Elements software (Nikon Instruments, Inc., Melville, NY, USA). Whole embryo z-stack images were captured in 1.675 µm steps, capturing several slices below and above the last seen Hoechst-stained nucleus to ensure all cells were captured.

Embryo z-stack images were analyzed using ImageJ software (version 1.54p, NIH, Bethesda, MD, USA). Briefly, a region of interest (ROI) outlining the full embryo was selected and used for all channels, across all the slices in the z-stack. BioVoxxel 3D Box threshold checker (ImageJ software, version 1.54p, NIH, Bethesda, MD, USA) was used to determine the optimal threshold for each channel and the same threshold was used for analyses of all samples and groups. Using particle analysis for all slices within the z-stack, the total fluorescent intensity was calculated using the sum of the integrated density divided by the sum of the area multiplied by the slice height in microns (1.675), expressed as the fluorescent intensity/µm^3^. Analysis of individual embryo slices was performed in a similar manner without multiplying by slice height.

For cell count comparisons, Hoechst channel z-stack images were analyzed using the Cell Counter plugin (ImageJ software, version 1.54p, NIH, Bethesda, MD, USA) which counts and marks each nucleus once so that the same cell is not counted twice across z-stacks.

### 2.10. Embryo Dissociation

For blastomere dissociation for scRNA-seq, freshly collected E4.5 embryos from either 1 or 2 dams (within the same experimental group) were pooled and cultured for 2–4 h then washed with pre-warmed 1 × DPBS with 0.04% BSA. Embryos were transferred to pre-warmed Acidic Tyrode’s solution (Sigma-Aldrich, St. Louis, MO, USA) for 1–2 min to remove the zona pellucida. Embryos were washed again, then transferred to pre-warmed dissociation media (Gibco^TM^ TrypLE^TM^ Select Enzyme, Thermo Fisher Scientific, Waltham, MA, USA) for 10 min at 37 °C followed by careful trituration by pipetting through a pulled glass capillary tube (Fisherbrand^TM^ blue top non-heparinized capillary tubes, Thermo Fisher Scientific, Waltham, MA, USA).

### 2.11. Single Cell-RNA Sequencing and Bioinformatics Processing

There are hurdles to scRNA of blastomeres because experiments typically require an input of 800 to 16,000 cells to meet the targeted retention of 500 to 10,000 viable cells, and the cell viability during processing is only 40–65%. E4.5 rat embryos have 25–30 cells on average, and the average number obtained per dam is 8–9 (200 to 270 cells per pregnant dam). Meeting minimum thresholds for cell input would require superovulation, mass breeding and/or longer culture and cryopreservation for batching, but this could introduce artifactual transcriptomic changes. To meet the minimum number of cells required for batch processing by the 10× Genomics scRNA-seq platform and still use freshly collected E4.5 embryos at the same developmental stage (blastocyst), we combined freshly dissociated blastomeres pooled from 1 or 2 dams within the same group and 1000 HEK-293T cells (ATCC^®^, Manassas, VA, USA). Post-run human sequences were then identified and removed from rat data during bioinformatics processing. Specifically, all cells were suspended in 1× DPBS with 0.04% BSA, which was mixed into a reaction containing reverse-transcription reagents and loaded onto Chromium Next GEM Chip G (Catalog number 1000120, 10× Genomics, Pleasanton, CA, USA). Reverse transcription, GEM generation and library preparation were performed using the Chromium Next GEM Single Cell 3’ Kit v3.1 (Catalog number 1000268, 10× Genomics, Pleasanton, CA, USA). The 10× Genomics Chromium Controller instrument was used to perform GEM (Gel beads-in-emulsion) generation. Full-length barcoded cDNAs were produced from poly-adenylated mRNA by reverse transcription within the GEMs. The transcripts were recovered from the GEMs, and the cDNAs were purified and amplified using 13 amplification cycles as per manufacturer recommendation. After clean-up of the amplification reaction, the concentration of cDNA solution was determined by a Bioanalyzer with the High Sensitivity DNA kit (Agilent, Santa Clara, CA, USA). To prepare sequencing libraries compatible with the Illumina platform, the entire cDNA reaction was fragmented, followed by end repair, A-tailing and adapter ligation. cDNA samples were amplified using dual sample indexes in the final step of library preparation, which enabled multiplexing during sequencing, then cleaned up, quantified, and sent to an outside vendor (Novogene, Durham, NC, UK) for sequencing.

Sequenced reads were aligned to the rat genome (rn7) using Cell Ranger (Version 9.0.1, 10× Genomics, Pleasanton, CA, USA). To assign the species of each cell to either human (HEK-293T) or rat, the output bam file from Cell Ranger was parsed into individual bam files for each unique barcode using the subsetbamlinux script (Version 1.1.0, 10× Genomics, Pleasanton, CA, USA), then BedTools (Version 2.29.1) was used to convert each bam into a fastq file. Each single cell fastq file was then aligned to either the rat or human genome using STAR (version 2.7.11b). The mapping rate to each genome was then used to determine the species of that cell, and human (HEK-293T) data was excluded from analyses. Cells were filtered to exclude those with more than 25% mitochondrial reads and retained if they had a detected unique gene count between 500 and 8000, ensuring the inclusion of cells with a sufficient number of expressed genes. Quality control steps were applied to remove low-quality cells while retaining ability to compare differences in genes encoded by the mitochondrial genome (mtDNA). All preprocessing was performed using Seurat v5.2.1 [47]. Data normalization was conducted using SCTransform version 0.4.2. During this step, the percentage of ribosomal gene expression was regressed out to minimize its potential confounding effect on downstream analyses. Cells were clustered based on shared expression profiles and visualized using UMAP embeddings.

### 2.12. Statistical Analysis

Statistical analyses of non-genomic data were performed using GraphPad Prism version 10.5.0 (GraphPad Software, Boston, MA, USA). Group and generational data were checked for normality, then comparisons were made using Student’s *t*-test or 1-way ANOVA with Mann–Whitney U or Kruskal–Wallis as appropriate for nonparametric tests. Significance was set at *p* ≤ 0.05.

For genomic data, differential expression analysis was performed using the MAST method implemented in FindMarkers, comparing certain conditions across all cells from CON and HF+DM groups within and across generations. A sub-set analysis of combined data included only cells expressing TE and ICM markers. Gene markers for each cell type were: Krt8, Gata2, Gata3, Wnt7b, Cdx2, Elf5 and Eomes for TE cells [48,49,50,51], and Pou5f1, Sox2 and Nanog for ICM cells [50,52,53,54,55]. An additional sub-set analysis was performed to differentiate sex as a biological variable. We used male gene markers expressed in early development (Eif2s3y, Usp9y, Kdm5d) to identify male embryonic cells. However, since very few sex-specific genes are expressed this early in development and because minor variations in timing of fertilization affect embryonic maturation and sex-specific gene expression, we could only confidently classify embryonic cells that expressed male genes, and all other cells were classified as “female/indeterminate”. Genes were considered significantly differentially expressed if they met a log fold-change threshold of >1 or <−1, with a *p*-value cutoff of 0.05. To investigate functional enrichment, the DEGs were analyzed using enrichGO from the ClusterProfiler v4.12.6 package [56]. This enabled the identification of enriched biological processes associated with gene expression changes across experimental conditions. Due to the nature of this study, embryos were collected from multiple generations that spanned across multiple years. To account for environmental confounding, genes that were significantly different (−1 ≥ log fold change ≥ 1 and *p*-value ≤ 0.05) between CON F2e and CON F3e were removed from DEG sets for all other comparisons.

Genomic data was also used to generate gene expression scores in which a set of genes related to a particular pathway or group were used to assess average expression across experimental groups. Three types of gene expression scores were used in this study. Mitochondria gene expression scores were used to estimate the mitochondria quantity through the expression of genes encoded by the mitochondrial genome (mtDNA). This gene set included Mt-nd1, Mt-nd2, Mt-nd3, Mt-nd4, Mt-nd4l, Mt-nd5, Mt-nd6, Mt-co1, Mt-co2, Mt-co3, Mt-atp6, Mt-atp8 and Mt-cyb. Oxidative stress response gene expression scores were used to determine the antioxidative response that cells had to oxidative stress. This gene set included Sod1, Sod2, Txnrd1, Txnrd2, Prdx1, Prdx2, Prdx3, Prdx5, Gpx1, Gpx2 and Gsr. Oxidative repair gene expression scores were also used to assess cellular repair response specific to base excision repair (BER), and included the gene set of Mutyh, Ogg1, Nthl1, Ung, Tdg, Mpg, and Polb.

The Broad Institute’s MitoCarta3.0 database [57] was used to compare DEGs that are localized to the mitochondrion. Since the database only contains mouse and human genomes, significantly different genes were analyzed using both mouse and human genome datasets. Results reported include conserved genes found in both. Genes found in common with the MitoCarta3.0 database were classified according to reported function. Seven categories were established, including metabolism, mitoribosomes and biosynthesis, cell signaling and cell fate, oxidative phosphorylation, oxidative repair, dynamism (fission/fusion), and structure/other functions.

All gene IDs and corresponding names described within the body text and figures of this manuscript are detailed in Table S13. High throughput sequencing data, including metadata, are deposited in the GEO database and may be accessed following the embargo period using the following accession number: GSE298842.

## 3. Results

### 3.1. Phenotypes of F0 Dams

Characteristics of the F0 control (CON) and high fat with diabetes (HF+DM) dams are detailed in Table 2. HF+DM F0 dams inadvertently weighed less at the start of diet (baseline) than CON F0 dams, which may be related to the age range of diet start (7–9 weeks). Despite this initial difference, HF+DM F0 dams gained approximately 4 times more weight prior to breeding (*p* < 0.0001) and were heavier at GD14 and P1 (*p* = 0.0008 and *p* = 0.0004, respectively). As expected, dams in both groups lost weight from GD14 to P1 which is related to delivery of pups and placentas. Whole blood glucose levels were not different after 4 weeks of diet (when breeding began) but increased dramatically in HF+DM dams following induction of diabetes at GD14 which led to a 4-fold higher average blood glucose from GD15 to GD21 and at P1 (*p* < 0.0001). Circulating triglyceride levels were significantly higher in HF+DM F0 dams prior to breeding and at P1 (*p* = 0.0131 and *p* < 0.0001, respectively). The number of placentations and pups/litter were not different between groups.

Taken together, our model simulates generational exposure to diet-mediated obesity complicated with gestational diabetes. HF+DM F0 dams expose F1 offspring to maternal overnutrition and hyperlipidemia from preconception to delivery combined with hyperglycemia in the last third of pregnancy when F1 gametogenesis occurs, effectively exposing primordial germ cells which will become F2 embryos (F2e).

### 3.2. Phenotypes of F1 and F2 Offspring

Characteristics of F1 female offspring used to create F2e are shown in Table 3. With pre-selection by birthweight, experimental HF+DM F1 females had similar birthweight and adult breeding weight as CON. This finding is not generalizable to all F1 offspring. Maternal age, measured at the start of pregnancy (post coital day 0.5), was different with HF+DM F1 dams being on average 4 weeks older than CON F1 dams (*p* < 0.0001). While this was significant, all dams remained below 31 weeks, which is well below predefined reports of advanced reproductive age in rats of 40 weeks [58,59]. Postnatal weight gain trended higher in HF+DM F1 offspring, but differences did not reach significance in this young adult subset likely due to birthweight stratification and a healthy lifestyle after birth. Ovaries from HF+DM F1 dams weighed more even when normalized to body weight (*p* < 0.0001), but there were no differences in the number of breeding days to achieve pregnancy or the number of E4.5 embryos collected when compared to CON. Neither glucose nor triglyceride levels were different at E4.5.

These findings highlight the point that metabolic health of the F1 parent is similar by group, so HF+DM F2e exposure to overnutrition is primarily during fetal primordial germ cell development in an adverse F0 in utero milieu (grandmaternal).

Characteristics of F2 female offspring used to produce F3e are shown in Table 4. Like F1, birthweight was not different by group. However, adult HF+DM F2 offspring weighed less than CON at breeding (*p* = 0.0062), even when age was similar. Like F1 dams, F2 HF+DM dams had higher ovary weights even when normalizing to body weight (*p* = 0.0146 and *p* = 0.0005, respectively). There were no differences in the number of breeding days to achieve pregnancy, but the number of embryos per litter was lower than CON (*p* = 0.0459). Additionally, HF+DM F2 dams had higher whole blood glucose levels at E4.5 compared to CON (*p* = 0.00533). Triglyceride levels were not different between groups.

These findings demonstrate that an adverse in utero milieu during fetal primordial germ cell development impacts adult weight, glucose metabolism, ovary size, and embryo number in grand-offspring (F2), a newly programmed phenotype that could impact F3e.

### 3.3. Multigenerational Effect of Diet and Diabetes on Embryo Morphology and Maturation

**Figure 2:**
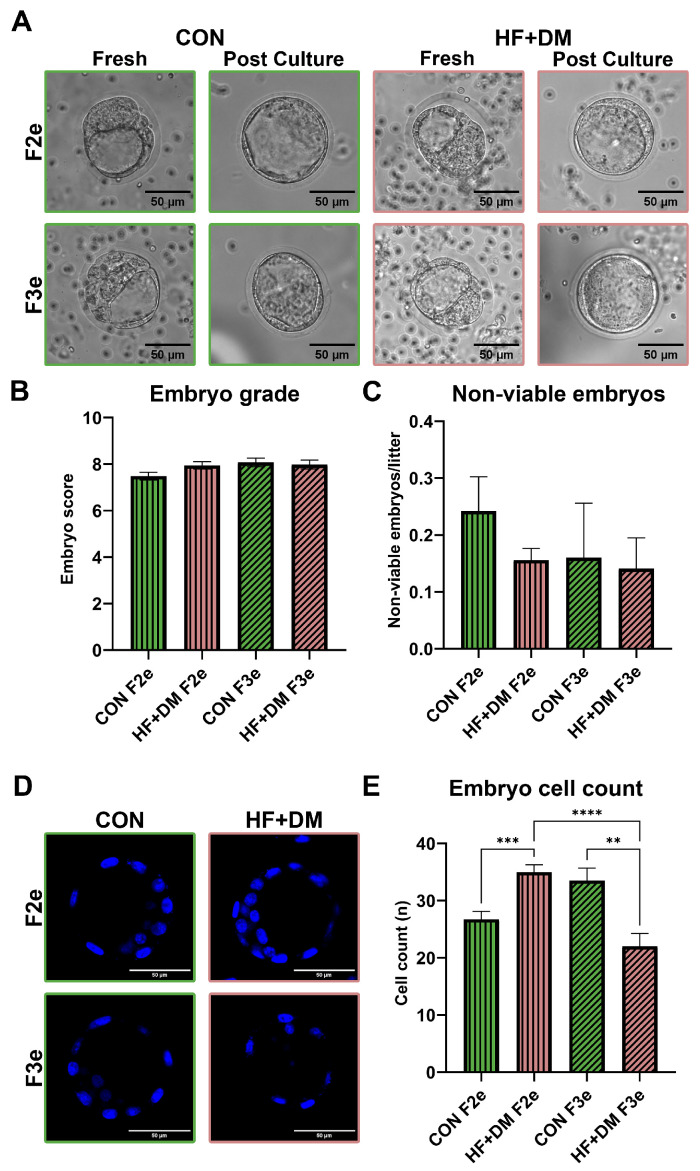
Group and generational embryonic morphology and maturation in E4.5 F2 and F3 embryos. Bright field images of embryos were captured following isolation on E4.5 (fresh) and after 2–4 h in culture (post-culture) for F2e and F3e in HF+DM and CON groups (**A**). Images from post-culture (**A**) were used to measure embryo grade which was converted to a numerical score for analysis (**B**). Embryo grades were also used to determine the percentage of embryos that were non-viable post-culture (**C**). Total embryo cell count was assessed by counting the number of cells stained with Hoechst from z-stack images of whole embryos (**D**,**E**). Bar graphs represent mean with SEM. F2e = F2 generation embryos. F3e = F3 generation embryos. N = 121–229 embryos/group for grading and viability, 6–28 embryos/group for cell counts. ** *p* < 0.01, *** *p* < 0.001, **** *p* < 0.00001.

Representative pre- and post-culture images of E4.5 embryos are shown in Figure 2A. There were no group-related differences in the embryo grades or viability, with most retrieved embryos having good expansion, TE and ICM quality at the E4.5 developmental stage (Figure 2B,C). HF+DM F2e had higher cell counts compared to CON F2e (*p* = 0.0009) but the opposite was found in the subsequent generation with HF+DM F3e having lower cell counts compared to both CON F3e and HF+DM F2e (*p* = 0.0064 and *p* < 0.0001, Figure 2D,E). Findings demonstrate that a HF+DM milieu during fetal primordial germ cell development increases F2e cell count which suggests advanced maturation or aging. However, the subsequent F3e may be impacted by a metabolic shift that leads to a compensatory delay in maturation.

**Figure 3:**
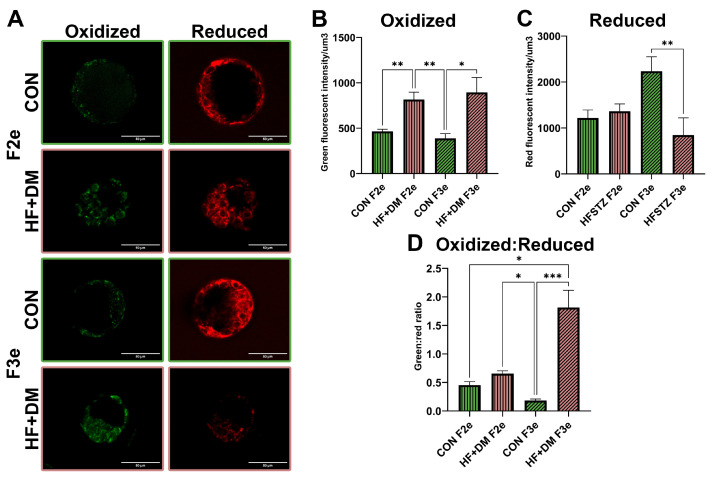
Group and generational evaluation of lipid peroxidation in E4.5 F2 and F3 embryos. BODIPY^TM^ 581/591 C11 probe was used to stain E4.5 embryos to assess lipid peroxidation. The middle slice of an individual embryo was captured (**A**) and used for analysis of oxidized lipids (green) (**B**), non-oxidized or reduced lipids (red) (**C**), and the oxidized:reduced (green:red) ratio (**D**). Bar graphs represent mean with SEM. N = 5–18 embryos/group. * *p* < 0.05, ** *p* < 0.01, *** *p* < 0.001.

Because reactive oxygen species (ROS) influence cell fate and maturation, we compared lipid peroxidation in HF+DM and CON F2e and F3e using BODIPY^TM^ C11, a lipid probe which fluoresces red in the reduced state and shifts to green upon oxidation. Representative images and group comparisons are shown in Figure 3. Both HF+DM F2e and HF+DM F3e had more oxidized lipids compared to their generational controls (*p* = 0.0058 and *p* = 0.0494, respectively, Figure 3B). HF+DM F3e also had less unoxidized (reduced) lipid than CON F3e (*p* = 0.0034, Figure 3C) making the ratio of oxidized to reduced lipid (green:red) ratio significantly higher than all other groups (*p* < 0.05, Figure 3D). Findings demonstrate that the HF+DM milieu during fetal primordial germ cell development increases lipid peroxidation in F2e and F3e, although F3e had less lipid accumulation overall. Taken together, fatty acid metabolism may be differentially regulated across generations.

### 3.4. Exposure-Mediated Transcriptomic Analysis of 2nd and 3rd Generation Embryos

**Figure 4:**
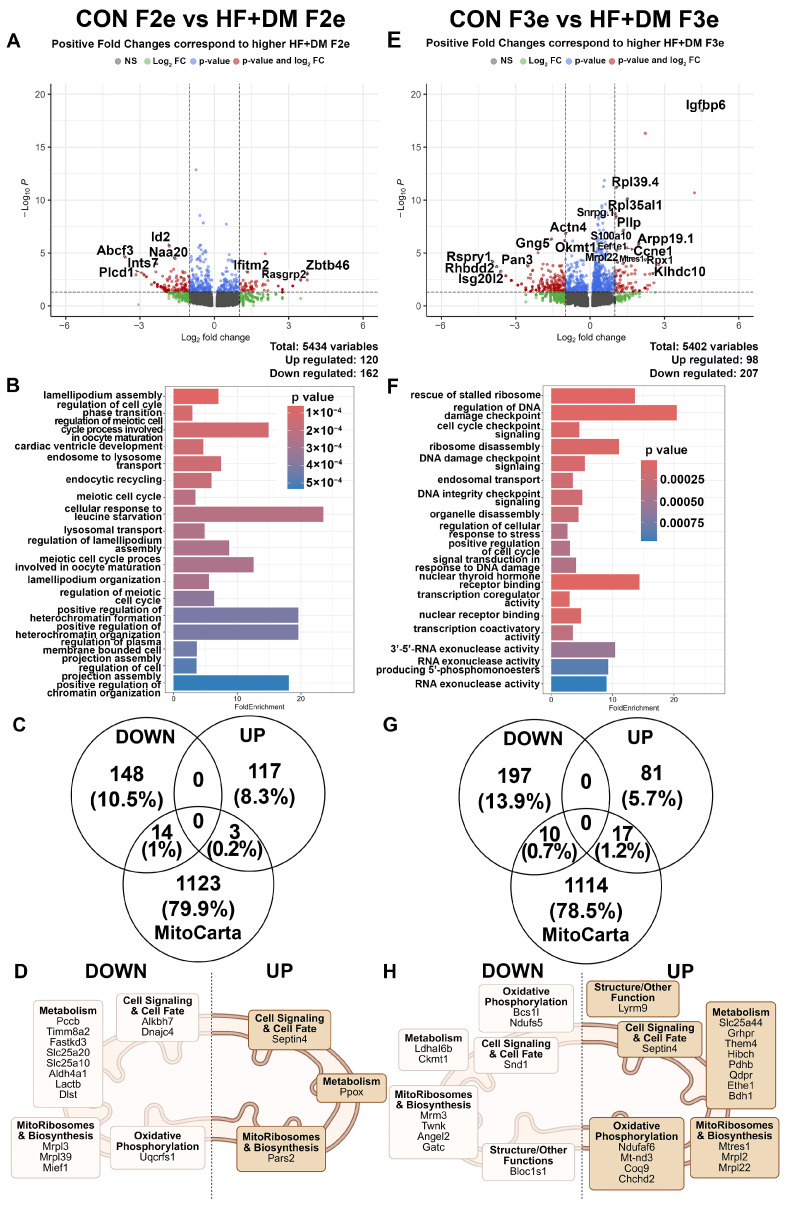
Exposure-mediated transcriptomic analysis of E4.5 F2 and F3 embryos. Differences between E4.5 embryos from CON F2e vs. HF+DM F2e results shown in the left column (**A**–**D**) and CON F3e vs. HF+DM F3e shown in the right column (**E**–**H**). Volcano plots (**A**,**E**) represent all DEGs and red dots show significance. Significant DEGs were assessed by pathway analysis (**B**,**F**) by fold enrichment of genes sorted by *p*-value. Significant up and downregulated DEGs were compared to the MitoCarta3.0 database (**C**,**G**) and were categorized by mitochondrial function (**D**,**H**). Mitochondria images (**D**,**H**) created in BioRender. Klein, Abigail. (2025) https://BioRender.com/av3p88u [66].

Group comparisons of scRNA-seq data from all embryonic cells (TE + ICM) within each generation are shown in Table 5 and Table 6 and Figure 4. Compared to CON F2e, HF+DM F2e had 282 DEGs with 162 downregulated and 120 upregulated genes (Figure 4A). The top 20 DEGs are detailed in Table 5 which includes the significantly downregulated inhibitor of DNA binding 2 (Id2), a transcriptional co-repressor of pluripotency and cell growth that influences stem cell differentiation, senescence, and apoptosis through insulin-like growth factor 1 receptor (IGF-1R) activation and downstream PI3K/Akt/mTOR pathways [60,61]. Downregulation could partially explain higher cell numbers in HF+DM F2e. Pathway analysis of DEGs are shown in Figure 4B with GO terms for biological processes on the *y*-axis and fold enrichment on the x-axis. Enrichment scores indicate how much a particular pathway is overrepresented. More statistically significant pathways are represented by red (lower *p*-values), while blue indicates *p*-values which are still statistically significant but less so. Highly enriched pathways (10–20-fold enrichment) include metabolism, regulation of heterochromatin formation and organization, chromatin organization, and meiotic cell-cycle regulation and processes, all which could impact oocyte and embryo maturation (Figure 4B). Highly significant (but less enriched) pathways include lamellipodium assembly and cardiac ventricle development pathways.

One biological interpretation of these key observations is that the F0 HF+DM milieu during fetal primordial germ cell development affects developmental pathways (meiosis), cell structure and transport through variable nutrient sensing and metabolic reprogramming and epigenetic modification (chromatin and heterochromatin). This is consistent with our findings of multigenerational programming in grand-offspring (F2).

To investigate the impact on mitochondrial health, DEGs were matched to the MitoCarta database (Figure 4C), and common genes were classified by their reported function (Figure 4D). There were 14 downregulated and 3 upregulated DEGs associated with mitochondria. Of interest, most mitochondria associated DEGs in HF+DM F2e are downregulated and cluster with metabolism, including fatty acid transport (Slc25a10, Slc25a20, Lactb) and oxidative phosphorylation (Uqcrfs1). Taken together, our findings suggest that a HF+DM milieu during fetal primordial germ cell development programs mitochondrial dysfunction and impaired metabolism to disrupt cell fate through epigenetic and signaling mechanisms in F2e.

In the F3 generation, there were 305 DEGs between CON and HF+DM embryos with 207 downregulated and 98 upregulated (Figure 4E). The top 20 DEGs are listed in Table 6. Highly enriched pathways include DNA damage response, genome integrity, nuclear thyroid hormone receptor binding, rescue of ribosome stalling or disassembly, and 3′-5′-RNA exonuclease activity pathways (Figure 4F). Additional highly significant pathways (low *p*-values) include cell-cycle checkpoint regulation. A biological interpretation of these key observations is that there is a shift from new epigenetic modification in F2e to adaptation to cellular stress, genome maintenance, RNA processing and ribosome regulation in F3e.

Matching DEGs from F3e to the MitoCarta database revealed 10 downregulated and 17 upregulated DEGs localized to mitochondria (Figure 4G,H). In contrast to the HF+DM F2e which had a downregulation of mitochondria and metabolism-related genes, the HF+DM F3e had an upregulation of mitochondria and metabolism genes. Specifically, there were multiple DEGs involved in the assembly and function of complex I of the electron transport chain (ETC) including NADH–Ubiquinone oxidoreductase subunit S6 (Ndufs6) and mitochondrially encoded NADH dehydrogenase 3 (Mt-nd3). Coenzyme Q9 (Coq9) was also upregulated. This gene encodes the predominant Coenzyme Q (CoQ) isoform in rats [62]. The CoQ complex serves as a potent mitochondrial antioxidant and facilitates electron flux from complex I and II to III. HF+DM F3e also had an upregulation of thioesterase super family 4 (Them4) which facilitates fatty acid oxidation, potentially explaining lower lipid accumulation in F3e. Taken together, our findings suggest that metabolic pathways improve in HF+DM F3e compared to HF+DM F2e, but opposite metabolic shifts influence nuclear to mitochondrial communication and cell growth, potentially contributing to delayed embryo maturation.

### 3.5. Exposure-Mediated Transcriptomic Analysis of 2nd and 3rd Generation Embryos in Trophectoderm and Inner Cell Mass Cells

**Figure 5:**
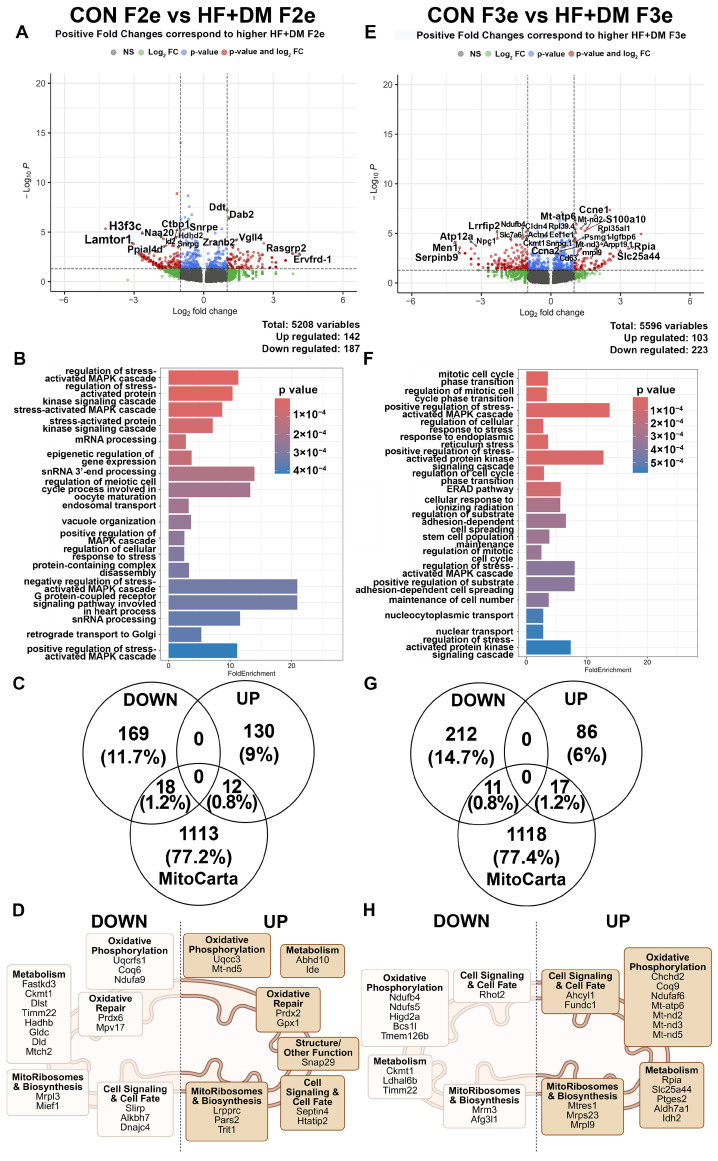
Exposure-mediated transcriptomic analysis of E4.5 F2 and F3 embryos in trophectoderm cells. Differences between E4.5 embryos from CON F2e vs. HF+DM F2e and CON F3e vs. HF+DM F3e in the trophectoderm cell type. Volcano plots (**A**,**E**) represent all DEGs between groups with red dots representing significance. Only significant DEGs were used for pathway analyses (**B**,**F**) by fold enrichment of genes sorted by *p*-value. Similarities between significant down- and upregulated DEGs and MitoCarta3.0 database (**C**,**G**) were categorized according to mitochondrial function (**D**,**H**). Mitochondria images (**D**,**H**) created in BioRender. Klein, Abigail. (2025) https://BioRender.com/s0dyit1 [67].

**Figure 6:**
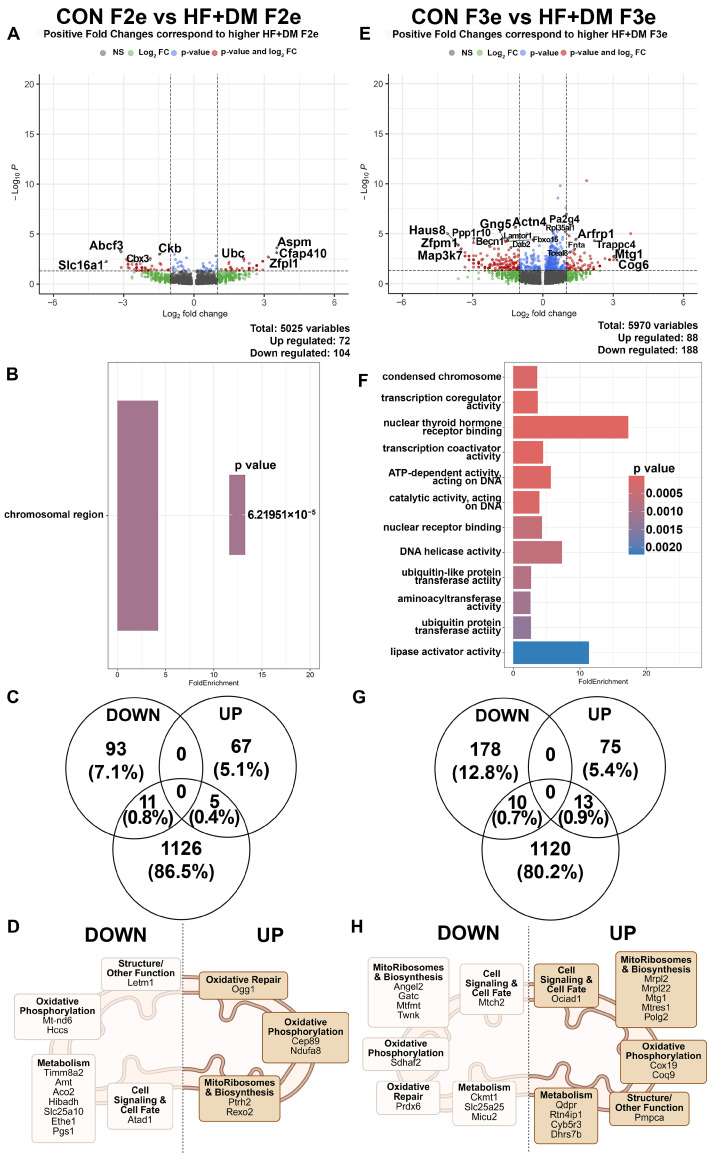
Exposure-mediated transcriptomic analysis of E4.5 F2 and F3 embryos in inner cell mass cells. Differences between E4.5 embryos from CON F2e vs. HF+DM F2e and CON F3e vs. HF+DM F3e in the inner cell mass cell type. Volcano plots (**A**,**E**) represent all DEGs between groups with red dots representing significance. Only significant DEGs were used for pathway analyses (**B**,**F**) by fold enrichment of genes sorted by *p*-value. Similarities between significant down- and upregulated DEGs and MitoCarta3.0 database (**C**,**G**) were categorized according to mitochondrial function (**D**,**H**). Mitochondria images created in BioRender. Klein, Abigail. (2025) https://BioRender.com/inj79ef [70].

At the E4.5 rat embryo stage, there are two major cell types, trophectoderm (TE) which gives rise to the placenta and inner cell mass (ICM) which gives rise to the embryo. Figure S2 shows a UMAP projection differentiating each cell type cluster. Group comparisons of scRNA-seq data within each generation are shown separately in Figure 5 for TE and Figure 6 for ICM.

For F2e TE, there were 329 DEGs with 187 downregulated and 142 upregulated in HF+DM compared to CON group (Figure 5A) The top 20 DEGs are listed in Table S3. Pathway analysis revealed a strong enrichment across positive and negative regulators of the stress-activated protein kinase and mitogen-activated protein kinase (MAPK) pathways that regulate cell fate through metabolic signaling (Figure 5B). Small nuclear RNA (snRNA) processing pathways involved in epigenetic regulation through post-transcriptional processes and meiotic processes are also significantly enriched.

Matching DEGs from F2e TE to the MitoCarta database identified 18 downregulated genes and 12 upregulated genes (Figure 5C,D). Some DEGs involved in oxidative stress response (Prdx6 and Mpv17) were downregulated, while others (Prdx2 and Gpx1) were upregulated. Like all embryonic cells (TE + ICM), genes involved in TE lipid metabolism (Mtch2 and Hadhb) were downregulated. Interestingly, Coq6, which encodes a key precursor in CoQ biosynthesis, was also downregulated. These findings suggest that a HF+DM milieu during fetal primordial germ cell development is associated with metabolic stress in TE that could cause abnormal implantation, placentation and placental dysfunction in the second generation.

In F3e TE, there were 326 total DEGs with 223 downregulated and 103 upregulated genes (Figure 5E). The top 20 genes are listed in Table S4. Three of the top genes were mitochondrially encoded genes (Mt-atp6, Mt-nd2 and Ndufb4) that were upregulated. Pathway analysis showed enrichment of genes associated with stress-activated MAPK pathways and cell-cycle regulation (Figure 5F). Matching DEGs from F3e TE to the MitoCarta database identified 11 downregulated and 17 upregulated DEGs (Figure 5G,H). Most mitochondria associated DEGs that were upregulated were involved in ETC complex assembly, oxidative phosphorylation, and metabolism (Mt-nd2, Mt-nd3, Mt-nd5, Ndufaf6, Mt-atp6, Chchd2, Coq9). However, there was also downregulation of genes involved in complex assembly (Ndufb4, Ndufs5, Hig2a, Bcs1l and Tmem126b). This split of up and downregulated genes involved in complex assembly may be related to an increase in mitochondrial quantity rather than function. Upregulated Mt-nd2, Mt-nd3, Mt-nd5, and Mt-atp6 encode proteins that serve as direct building blocks for complex 1 (Mt-nd2, Mt-nd3, Mt- nd5) and complex V (Mt-atp6) assembly. Upregulated Ndufaf6 is not a complex subunit but it regulates the synthesis of Mt-nd1 needed for biogenesis [63]. In contrast, downregulated complex I genes include Ndufb4, which interacts with complex I and III during respirasome assembly [64], and Ndufs5, which modifies complex I function through post-translational modification [65]. Of interest, FUN14 domain-containing 1 (Fundc1) was upregulated, which is involved in mitochondrial quality control through mitophagy. Taken together, our findings suggest HF+DM F3e TE have a responsive increase in mitochondrial quality control and replication (mitochondria quantity) alongside downregulation of genes involved with respiration (mitochondria function), possibly in response to mitochondrial dysfunction and ROS.

Similar analyses were performed for ICM. In HF+DM F2e compared to CON F2e, there were 176 DEGs with 104 down- and 72 upregulated genes (Figure 6A). The top 20 DEGs are listed in Table S5. Top DEGs fell into the significantly enriched GO term of ‘chromosomal region’ which refers to cellular components including centromeres, telomeres, promoters and enhancers, rather than to a dynamic biological pathway (Figure 6B). A MitoCarta database query identified 11 down- and 5 upregulated genes in HF+DM F2e ICM (Figure 6C,D). Like all embryonic cells (TE + ICM), most of the mitochondria associated DEGs in HF+DM F2e were downregulated and clustered with metabolism including Mt-nd6, one of the top 20 genes from this comparison. Of interest, HF+DM F2e ICM have an upregulation of 8-oxoguanine DNA glycosylase (Ogg1), which is crucial for repairing guanine oxidation (8-oxodG).

In F3e ICM, there were 276 DEGs with 188 down- and 88 upregulated genes (Figure 6E). The top 20 genes are listed in Table S6. Pathway analysis revealed enrichment of pathways involved in nuclear thyroid hormone receptor binding and lipase activation (Figure 6F). A MitoCarta query showed 10 downregulated and 13 upregulated mitochondria associated genes (Figure 6G,H). Like HF+DM F3e TE, Coq9 was also upregulated in ICM, possibly in response to oxidative stress.

In summary, HF+DM F2e TE and ICM cells both had downregulated mitochondria and metabolism associated genes, but F2e ICM seemed to have fewer DEGs impacted by the HF+DM milieu during fetal primordial germ cell development than F2e TE. This could indicate a partially protective effect for organogenesis compared to placentation. Interestingly, F3e had more DEGs in ICM than F2e. HF+DM F3e TE and ICM had upregulated genes associated with mitochondria and metabolism. Taken together, our findings demonstrate that even without direct exposure, the third-generation embryos experienced novel transcriptomic changes related to DNA repair and mitochondrial quality control that could impact the metabolic health and development of both the placenta and F3 offspring.

### 3.6. Generational Transcriptomic Analysis in Exposed Embryos

Differences between exposed HF+DM F1 and HF+DM F2 dams used to generate F2e and F3e are shown in Table S7. HF+DM F2 dams had higher birthweights but lower adult weights at the time of breeding (*p* = 0.0443 and *p* = 0.0016, respectively). As previously noted, HF+DM F1 dams were inadvertently older than HF+DM F2 dams at the start of pregnancy (*p* < 0.0001). Regardless, all dams remained less than 31 weeks which is below the commonly defined advanced reproductive age of 40 weeks [58,59]. Ovary weights, even when normalized to body weights, were lower in HF+DM F2 dams (*p* < 0.0001 and *p* = 0.0106, respectively). There were no generational differences in breeding days, embryos/litter or glucose; however, HF+DM F2 dams had lower triglycerides at E4.5 (*p* = 0.0143).

**Figure 7:**
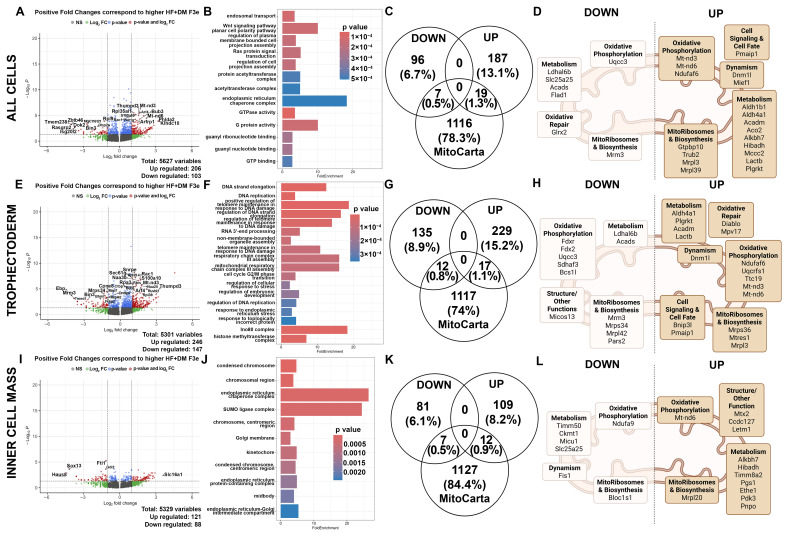
Generational transcriptomic analysis in HF+DM E4.5 embryos. Differences between E4.5 embryos from HF+DM F2e vs. HF+DM F3e in total cells (**A**–**D**), trophectoderm cells (**E**–**H**), and inner cell mass cells (**I**–**L**) were compared. Volcano plots (**A**,**E**,**I**) represent all DEGs between groups with red dots representing significance. Significant DEGs were used for pathway analyses (**B**,**F**,**J**) by fold enrichment of genes sorted by *p*-value. Similarities between significant down and upregulated DEGs were compared to MitoCarta3.0 (**C**,**G**,**K**) and were categorized by mitochondrial function (**D**,**H**,**L**). Mitochondria images created in BioRender Klein, Abigail. (2025) https://BioRender.com/4b93xep [71].

Generational differences between HF+DM F2e and HF+DM F3e were analyzed to determine whether transcriptomic variations normalized, persisted or were compounded across generations. Data is shown in Figure 7. In all HF+DM exposed embryonic cells (TE + ICM), there were 309 DEGs with 103 downregulated and 206 upregulated genes from F2e to F3e (Figure 7A). The top 20 DEGs are listed in Table 7 and included genes involved in RNA/ribosome biogenesis (Polr1g, Thumpd3), protein quality control (Hspa14, Pfdn4, Naa20), trafficking (Arf4, Arfrp1, Ccz1b, S100a10), mitotic checkpoints (Bub3) and development (Phlda2, Rac1). There was also upregulation of mitochondrially encoded complex I genes (Mt-nd3, Mt-nd6) in HF+DM F3e. Pathway analysis showed high enrichment (10–20-fold) of endoplasmic reticulum chaperone complexes which play a critical role in protein folding and quality control. Additional highly significant pathways (low *p*-value) included GTPase and G protein activity, endosomal transport and Wnt signaling (Figure 7B). Matching to the MitoCarta database identified 7 downregulated and 19 upregulated.

Genes in common (Figure 7C,D). As expected, most of the upregulated genes are involved in metabolism, including Ndufaf6, Mt-nd3, and Mt-nd6, which were the three subunits of complex I that were also higher in HF+DM F3e compared to generational control. Acyl-CoA dehydrogenase medium chain (Acadm), which encodes for an enzyme crucial for the beginning steps of fatty acid oxidation of medium chain fatty acids, and Dnm1l, which is important in mitochondrial fission, also increased from F2e to F3e.

For TE cells, there were 393 DEGs with 147 downregulated and 246 upregulated in HF+DM F3e (Figure 7E). The top 20 genes are listed in Table S8. This includes upregulation of replication protein A3 (Rpa3), a subunit of the RPA complex involved in DNA damage control that also influences cell fate through the PI3K/Akt and mTOR pathways [68]. Highly enriched pathways from F2e to F3e included DNA repair, maintenance and replication, chromatin remodeling (Ino80 complex), and mitochondrial respiratory chain complex 3 assembly (Figure 7F). A MitoCarta database query identified 12 downregulated and 17 upregulated mitochondria associated genes (Figure 7G,H) including Ndufaf6, Mt-nd3, Mt-nd6 and Dnm1l, and Acadm, all which were also upregulated in the total cell comparison. Additionally, HF+DM F3e TE cells had an upregulation of genes involved in apoptosis (Diablo and Bnip3l) and mitochondria DNA maintenance (Mpv17) compared to HF+DM F2e.

For ICM cells, there were 209 DEGs with 88 downregulated and 121 upregulated (Figure 7I). The top 20 genes are listed in Table S9. Pathway analysis demonstrated high enrichment of genes involved in the endoplasmic reticulum chaperone complex (similar to total cells) and sumoylation pathways that facilitate DNA and protein repair after oxidative stress and influence centromere function for the cell cycle (Figure 7J). There were 7 downregulated and 12 upregulated genes found in common with the MitoCarta database (Figure 7K,L). HF+DM F3e ICM had an upregulation of Mt-nd6 (but not Ndufaf6, Mt-nd3 or Dnm1l). Acadm was not different in ICM, but Ccdc127 which is involved in lipid droplet regulation was upregulated [69]. Pyruvate dehydrogenase kinase 3, Pdk3, was also upregulated in the ICM cells across generations. This encodes a subunit of the pyruvate dehydrogenase complex which is important for converting pyruvate to acetyl-CoA during glycolysis. HF+DM F3e ICM also had a downregulation of Ndufa9 (complex I) and Fis1 (mitochondrial fission protein).

Overall, across generations, the HF+DM F3e had upregulated stress response, damage repair, mitochondria biogenesis and metabolic pathways, likely an adaptive response to rescue metabolic impairment found in F2e. Given this transcriptional reversal, it is not surprising that these adaptive cellular responses impacted embryo growth and development in opposing directions from advanced to delayed maturation.

**Figure 8:**
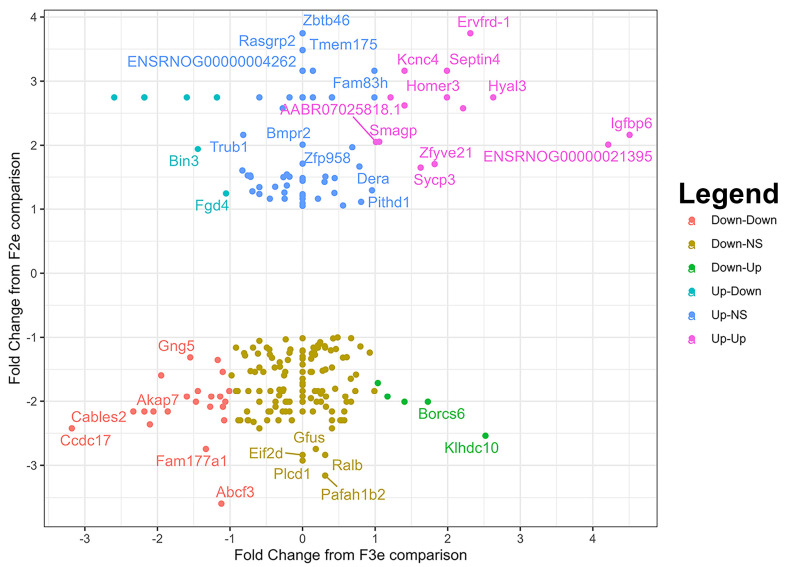
Gene fold changes from E4.5 F2 to F3 embryos. Genes with a significant fold change from E4.5 embryos from HF+DM F2e vs. CON F2e comparison (y-axis) compared to genes with a significant fold change from E4.5 embryos from HF+DM F3e vs. CON F3e comparison (x-axis). The color of dot represents whether a gene was down- or upregulated in the F2e and whether that same gene was also upregulated, downregulated, or no longer significant (NS) in F3e. See “Legend” on right side of graph for color code.

This fluctuating generational impact from HF+DM became even more apparent by plotting the fold change in HF+DM F2e vs. CON F2e on the y axis by HF+DM F3e vs. CON F3e on the x axis as shown in Figure 8. This highlights the directionality of DEGs across generations. Specifically, genes that were highly upregulated in F2e then switched to downregulated in F3e included Bin3 and Fgd4, both involved in actin-mediated cytokinesis and cell division. Genes that were highly downregulated in F2e but switched to upregulated in F3e included Klhdc10 and Borcs6. Klhdc10 regulates apoptosis under oxidative stress [72], and Borcs6 is involved in embryonic lysosomal trafficking [73]. Both are important regulators of quality control and cell fate under stress. Conversely, DEGs with similar directionality across generations demonstrate stable or compounded transgenerational programming. Several genes were upregulated in both generations including Septin4, Igfbp6, and Ervfrd-1, as well as Kcnc4, Homer3, Smagp, Zyfyve21, and Sycp3. Specifically, Septin4 encodes a pro-apoptotic protein that binds with Parp1 to regulate mitochondria and oxidative stress-induced cell death [74,75,76]. Insulin-like growth factor binding protein 6 (Igfbp6) encodes a protein that inhibits insulin-like growth factor 2 (Igf2) to play a crucial role in cell proliferation during embryonic development [77,78]. Ervfrd-1, also known as Syncytin-2, plays a critical role in trophoblast cell fusion which is necessary for trophoblast syncytialization; overexpression is associated with placental abnormalities and early pregnancy loss [79,80,81]. Abcf3, Gng5, Akap7, Cables2, Ccdc17, and Fam177a1 were all downregulated in both generations. Of particular interest, Cables2 is a cyclin-dependent kinase involved in p53-dependent and independent apoptosis, and it plays an important role in early embryo development [82]. In summary, HF+DM led to similar and divergent transcriptomic changes in preimplantation embryos. Much of the transcriptomic variation in F2e was transient, which protects unexposed F3e generations from programming effects; however, other genomic variations persisted or expanded across generations, and they also regulate cell fate. This data suggests that HF+DM not only impacts transcriptional regulation of embryonic growth and development for one, but for multiple generations, even without direct exposure.

### 3.7. Exposure-Mediated and Generational Transcriptomic Analysis of 2nd and 3rd Generation Embryos in Female/Indeterminate Cells

Because sex is a biological variable that impacts the developmental origins of health and disease, we investigated transcriptomic differences in female embryos to determine whether the enriched pathways were similar to grouped sexes. During preimplantation embryo development, sex-specific gene expression is highly variable due to dynamic processes of methylation, X chromosome inactivation and imprinting. These processes are not only cell-type specific, but they also change quickly across embryo maturation and can be reversed in the ICM [83,84]. Excluding genes that are not yet expressed at E4.5 or are impacted by X-inactivation leaves very few sex-specific genes that are measurable at this early stage. Using current literature to guide us, we identified three male-specific, Y chromosome genes (Eif2s3y, Usp9y, Kdm5d) that were expressed within 30% of our E4.5 dataset. These genes are all located on the Y chromosome and are reportedly expressed at this stage [85,86]. We defined the remaining 70% of cells to be female and/or indeterminate. This is because we cannot be certain these are all truly female, or rather, some may not yet express male gene markers. We completed a sub-set analysis using only these female/indeterminate cells to further define multigenerational outcomes specific to inheritance through the female germline and to separate sex-related transcriptomic differences.

In the F2 generation, there were 177 downregulated and 67 upregulated genes in HF+DM F2e female/indeterminate cells compared to their generational control (Figure S3). In relationship to the combined data, female/indeterminate cells had a similar number of downregulated genes, but much fewer upregulated genes. The top 20 DEGs are listed in Table S10. Five of these top 20 DEGs were unique to female/indeterminate cells. These included Rpl38.1, Ttc4, Borcs8, Uba2, and Sf3b4, all important to cell quality control mechanisms. Pathway analysis (Figure S3) established similar enrichment of cell-cycle regulation-related pathways compared to total cells (Supplementary File S2 Excel); however, RNA processing pathways were uniquely enriched in female/indeterminate cells. Overall, HF+DM F2 female embryos have enhanced RNA processing and fewer upregulated DEGs than combined sex comparison, possibly because oogonia have better intrinsic cellular defenses to combat DNA damage as they are protected by supporting cumulus and granulosa cells, as well as antioxidant-rich follicular fluid in the ovary [87].

In the F3 generation, there were 146 down and 104 upregulated genes in HF+DM F3e compared to CON F3e in female/indeterminate cells (Figure S3). This was similar to the combined sex comparison. The top 20 DEGs are listed in Table S11. There was only 1 gene in the top 20 DEGs that was not also found to be significant in the combined sex comparison, SHC binding and spindle associated 1 (Shcbp1). Shcbp1 was upregulated in HF+DM F3e compared to generational female/indeterminate controls; and is necessary for proper meiosis and cell division [88]. In the pathway analysis, most enriched pathways in female/indeterminate cells were also enriched in the combined sex comparison including pathways related to damage/stress response, RNA quality control, cell-cycle regulation, maintenance of cellular homeostasis, and prevention of cellular/RNA breakdown which can be related to gene expression regulation (Supplementary File S2 Excel). Taken together, these findings suggest that HF+DM F3 female/indeterminate embryos have similar transcriptomics as HF+DM F3 exposed male cells.

Generational transcriptomic differences between exposed HF+DM F2e and HF+DM F3e in female/indeterminate cells were also evaluated (Figure S3). There were 76 downregulated and 225 upregulated genes from F2 to F3 generations, a quantity that is similar to the combined sex comparison. Of the top 20 genes (Table S12), there were 4 novel DEGs unique to female/indeterminate cells, including Asxl1, Mta3, Tmem59, and Commd2 which regulate cell fate through epigenetic (Asxl1, Mta3) and cell signaling pathways that are important during development (Tmem59, Commd2). In the generational pathway analysis, there were both similar and uniquely enriched pathways in female/indeterminate cells compared to the HF+DM exposed cells from the combined sex comparison (Figure S3, Supplementary File S2 Excel). Similarly enriched pathways included cell projection/structure/cilium pathways, cellular homeostasis and quality control, and RNA processing, whereas uniquely enriched pathways included damage/stress response, chromosome organization/methylation and embryonic development pathways. Interestingly, several newly identified pathways in female/indeterminate cells were also enriched in TE, but not ICM from the combined sex comparison. Overall, these findings suggest that females may have a more robust adaptation to cellular stress and epigenetic regulation.

To further investigate inheritance through the female germline, we assessed directionality of expression across generations in the female/indeterminate subset. Specifically, we were interested in identifying DEGs that were down- or upregulated by F0 HF+DM exposure and that persisted or compounded in expression across generations. Unique to the female/indeterminate subset, tetratricopeptide repeat domain 4 (Ttc4) was downregulated in both HF+DM F2e and HF+DM F3e compared to generational controls. Ttc4 acts as a co-chaperone of Hsp70 and Hsp90, two heat shock proteins activated during stress response to maintain protein quality control [89,90]. The other gene that compounded across generations was Igfbp6, which was upregulated in both HF+DM F2e and HF+DM F3e. This gene was previously identified in our combined sex comparison, but again showed a significant persistent effect across generations. It was further upregulated in HF+DM F3e female/indeterminate cells not only compared to generational controls like combined sexes but also compared to HF+DM F2e in female/indeterminate cells. This further highlights the importance of Igfbp6 across both female and male embryo, but may be more pronounced in females. Taken together, these 2 DEGs serve as important candidates for regulating transgenerational inheritance through the female germline.

There were additional DEGs that were unique to the female/indeterminate subset whose expression changed directionality across generations, which suggests ability to escape programming or adapt across generations. BLOC-1 related complex subunit 8 (Borsc8), splicing factor 3B subunit 4 (Sf3b4), and ASXL transcriptional regulator 1 (Asxl1) had a down-up pattern of dysregulation compared to controls. Borcs8 plays a key role in lysosomal function [91], which is important in autophagy. Sf3b4 regulates mRNA splicing and is downregulated in states of stress to effect transcription and translation [92]. Lastly, Asxl1 is a transcriptional regulator involved in chromatin remodeling, specifically inactivation of H3K27me3-mediated gene silencing [93]. Directionality data supports pathway analysis and informs sex-specific mechanisms of transgenerational inheritance and programming escape.

### 3.8. Mitochondria and Oxidative Stress in F2 and F3 Embryos

**Figure 9:**
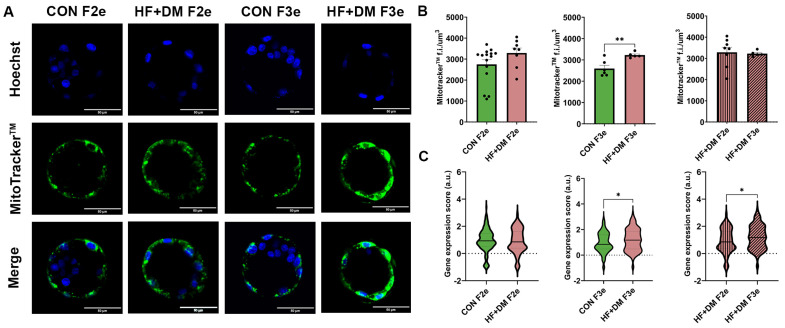
Group and generational mitochondria evaluation in E4.5 F2 and F3 embryos. Post-culture E4.5 embryos were stained with Hoechst and MitoTracker^TM^, then full embryo z-stack images were acquired on a confocal microscope (**A**). Analysis of fluorescent intensities (f.i.) was performed using ImageJ (version 1.54p) (**B**). Gene expression score was generated using 13 protein-encoding genes from the mitochondrial genome from scRNA-seq data (**C**). Bar graphs represent mean with SEM and violin plots represent median with quartiles. Black dots (**B**) represent individual embryos. N = 5–18 embryos/group for live cell imaging. * *p* < 0.05, ** *p* < 0.01.

To examine the impact of transcriptomic variation on mitochondria and oxidative stress, we stained and imaged E4.5 embryos and quantified mitochondria and ROS fluorescent intensity across full z-stacks and performed group comparisons (Figure 9). By staining, there was no difference in mitochondria quantity in HF+DM F2e compared to CON F2e. However, in the third generation, mitochondria quantity was higher in HF+DM F3e compared to CON F3e (*p* = 0.0063, Figure 9A,B). Imaging data affirmed scRNA-seq mitochondria gene expression scores which include 13 genes encoded by the mitochondrial genome: Mt-nd1, Mt-nd2, Mt-nd3, Mt-nd4, Mt-nd4l, Mt-nd5, Mt-nd6, Mt-co1, Mt-co2, Mt-co3, Mt-atp6, Mt-atp8 and Mt-cyb. Specifically, HF+DM F3e had a higher mitochondria gene expression score than CON F3e and HF+DM F2e (*p* = 0.0108 and *p* = 0.0407, Figure 9C).

**Figure 10:**
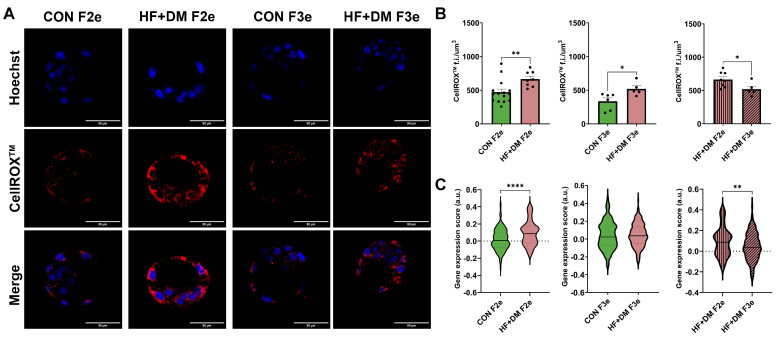
Group and generational oxidative stress evaluation in E4.5 F2 and F3 embryos. Post-culture E4.5 embryos were stained with Hoechst and CellROX^TM^, then full embryo z-stack images were acquired on a confocal microscope (**A**). Analysis of fluorescent intensities (f.i.) was performed using ImageJ (version 1.54p) (**B**). Gene expression score using scRNA-seq data was generated using genes involved in response to oxidative stress (**C**). Bar graphs represent mean with SEM and violin plots represent median with quartiles. Black dots (B) represent individual embryos. N = 5–18 embryos/group for live cell imaging. * *p* < 0.05, ** *p* < 0.01, **** *p* < 0.0001.

Mitochondria are the primary producers of ROS, especially when they are dysfunctional. Excess ROS can incite oxidative stress and DNA damage, a phenotype that was predicted by transcriptomic data. To validate this finding, we stained E4.5 embryos with CellROX^TM^ which detects ROS. Representative images are shown in Figure 10. Image analyses demonstrated more ROS in both HF+DM F2e and HF+DM F3e embryos compared to generational controls (*p* = 0.0098 and *p* = 0.0264, Figure 10B). Although it was significantly higher across two generations, ROS staining decreased from HF+DM F2e to HF+DM F3e (*p* = 0.0396). We used a similar method of gene expression scoring from scRNA-seq data to estimate cellular responses to oxidative stress. This subset included the following genes: Sod1, Sod2, Txnrd1, Txnrd2, Prdx1, Prdx2, Prdx3, Prdx5, Gpx1, Gpx2 and Gsr. In alignment with embryo imaging, HF+DM F2e had a higher oxidative stress gene expression score compared to both CON F2e and HF+DM F3e (*p* < 0.0001 and *p* = 0.0061, Figure 10C).

Oxidative stress can incite oxidative damage repair. To examine this cellular response to ROS, we generated an oxidative repair gene expression score. This subset included genes involved in base excision repair including: Mutyh, Ogg1, Nthl1, Ung, Tdg, Mpg, and Polb. We found that HF+DM F2e had a higher oxidative repair gene expression score than the generational control, but HF+DM F3e was not different than controls (Figure S4). Overall, embryo staining affirmed transcriptomic findings that a HF+DM milieu incites oxidative stress without increasing mitochondria abundance in HF+DM F2e, but an adaptive increase in mitochondria quantity and damage repair mechanisms improved metabolism and ROS production in the subsequent HF+DM F3e generation.

## 4. Discussion

In the present study, we used a rat model of overnutrition plus late gestation diabetes to elucidate transgenerational transcriptomic consequences for F2 and F3 embryos. We have already reported the effects of developmental programming on first generation offspring and identified specific epigenomic and transcriptomic alterations that contributed to lifelong cardiometabolic consequences [30,31,32,33,34,35,36,37,38]. The goal of this study was to determine whether there were lasting impacts on preimplantation embryos across multiple generations. Our model uniquely mimics generational exposure to a common comorbid state of obese pregnancy complicated by gestational diabetes. Specifically, F1 offspring and its developing primordial germ cells, which will become the F2 generation, are exposed to maternal hyperglycemia, hyperlipidemia and fetal hyperinsulinemia during the last trimester of F0 pregnancy. We limited confounding postnatal variables by birthweight stratification, early cross-fostering of equal litter sizes to unaffected dams, and weaning pups to control diets through breeding. Using this strategy, F3e had no direct prenatal or postnatal exposures beyond transgenerational programming. We hypothesized that HF+DM F2e would have transcriptional perturbations that impact metabolic health and that some, but not all of these transcriptional changes would be mitigated in the third generation due to the lack of direct exposure. As expected, there were many DEGs in the HF+DM-exposed second generation. The enriched pathways alongside embryo staining demonstrated mitochondrial dysfunction, oxidative stress and epigenetic activation that led to an advanced aging embryonic phenotype. Across generations, many DEGs were no longer significantly up or downregulated in HF+DM F3e. This highlights the ability to reset and escape programming at the primordial germ cell or zygote stage. As expected, we also identified some sustained and compounded gene expression patterns which are evidence for persistent programming into the third generation. Unexpectedly, HF+DM F3e expressed many novel DEGs in pathways related to cell stress response. These could be activated directly by maternal inheritance of dysfunctional mitochondria or by an indirect response to the F2 maternal phenotype (lower weight and higher blood glucose at breeding). Regardless, the HF+DM F3e enriched pathways and embryo staining predict an exaggerated metabolic shift towards oxidative phosphorylation and cellular homeostatic responses to cell stress that resulted in a unique, underdeveloped embryonic phenotype and fewer embryos, a marker of infertility.

For the second generation, this study established that obese, diabetic pregnancy not only impacts the F1 offspring but also the subsequent F2 grand-offspring. We also showed that programming effects are already established at the preimplantation embryo stage and may occur even when there is a healthy postnatal environment, and maternal phenotypes are similar (body weight, glucose and lipid levels). Specifically, we found that HF+DM F2e, which were exposed as primordial germ cells, had evidence of mitochondrial dysfunction, impaired lipid metabolism, chromatin remodeling and epigenetic activation impacting metabolic signaling and cell fate. Mitochondrial dysfunction can shift cellular metabolism from oxidative phosphorylation to glycolysis which drives stem cell proliferation through cell signaling pathways. Mitochondrial dysfunction also increases ROS generation, triggering early differentiation and aging, as well as DNA damage which incites further transcriptional dysregulation. This study identified specific transcriptional regulators that likely play a role. Id2, a Helix-Loop-Helix transcriptional co-repressor that is crucial for maintaining a balance between stemness and differentiation [94] was markedly downregulated in HF+DM F2e. There is good evidence that Id2 expression is regulated by DNA methylation, and that hypermethylation in promotor regions decreases Id2 expression triggering advanced differentiation [95,96]. Several other genes that are known to shift cellular metabolism and alter cell fate through PI3K/Akt and mTOR dysregulation were also downregulated (Pccb, Aldh4a1, Lactb) [97,98,99]. This is interesting given our previous findings that F1 newborns had decreased activation of the PI3K/Akt pathway in myocardium [34] and lung vasculature which contributed to phenotypic cardiomyopathy and pulmonary hypertension at birth [35]. Metabolic shifts and oxidative DNA damage can also influence transcription via epigenetic modification of histones which change chromatin structure. In this study, HF+DM F2e had an enrichment of chromatin remodeling pathways and a female-specific downregulation of Asxl1, which moderates H3K27me [93]. We previously reported a HF+DM-mediated increase in H3K27me3 repressive marks (89%) alongside a decrease in H3K4me3 and H3Ac activating marks (33% and 53%) at promoter sites that dysregulated cardiometabolic health in F1 offspring [33]. It is likely that fetal primordial germ cells exposed to the same adverse in utero environment as their F1 parents have similar epigenetic programming. Although we have not yet reported the long term metabolic consequences in adult grand-offspring, we predict that early embryonic activation of stem cell proliferation, exhaustion or depletion of stem cell pools, and epigenetic drift will impact the lifelong cardiometabolic health of the second generation [100,101,102,103,104,105,106,107,108,109,110,111].

This study also established that obese and diabetic pregnancy has lasting transgenerational effects to the third generation. We found that transcriptomic dysregulation in F3 is already established at the preimplantation embryo stage and occurs without a second hit phenomenon. In contrast to the second generation, HF+DM F3e have enrichment of pathways involved in mitochondrial biogenesis, oxidative phosphorylation, DNA repair, and cell-cycle checkpoint regulation. Imaging confirmed higher mitochondria quantity and less lipid accumulation, even though lipid peroxidation remained higher than generational controls. These adaptive shifts in metabolism were associated with a lower cell number and fewer embryos, signs of delayed embryo maturation that was in sharp contrast to the advanced aging seen in HF+DM F2e. Adaptive transcriptomic responses included upregulation of genes involved in mitochondria protein synthesis and biogenesis (Gtbp10, Trub2, Mrpl3, Mrpl39) and mitochondrial quality control (Dnm1, Mief1). This could explain why HF+DM F3e had more mitochondria. HF+DM F3e also had enrichment of pathways that mitigate oxidative stress. This included an upregulation of Coq9 expression which encodes the mitochondrial antioxidant, ubiquinone or CoQ. Interestingly, we previously reported a downregulation of Adck3 in HF+DM-exposed F1 offspring hearts [34]. Adck3 is also involved in CoQ biosynthesis and decreased expression could explain impaired oxidative phosphorylation and oxidative stress that caused cardiometabolic disease in F1 offspring. It is likely that F2 fetal primordial germ cells exposed to the same adverse in utero environment are affected too, but that the unexposed F3e adapts by increasing CoQ synthesis. Overall, the predicted developmental effect for HF+DM F3e is a premature shift from glycolytic to lipid metabolism, alongside oxidative stress, DNA damage repair and exaggerated stress responses that impedes proliferation and results in early differentiation or senescence [112,113,114]. Taken together, we propose that the (unexposed) third generation could be the first generation to begin improving mitochondrial function, and that CoQ supplementation could be a potential therapeutic strategy to mitigate programming effects from an obese and diabetic pregnancy.

Another strength of our study was sex-specific transcriptomic analyses. Using this approach, we found HF+DM F2e female cells had fewer upregulated DEGs. While similar pathways were enriched, female embryos had a unique enrichment of RNA processing pathways compared to combined sexes. Interestingly, chromatin formation/organization and cardiac development pathways were enriched when sexes were combined but were not highly enriched in females alone. These are interesting findings given the prominence of sexual dimorphism that exists in the developmental origins of health and disease (DOHaD) field, including that males have a higher susceptibility to cardiometabolic disease [115]. Others have suggested that males have an exaggerated response to in utero stress, thus have a higher susceptibility to adult programmed disease [116,117]. Encouraging findings were that many of the enriched pathways identified in HF+DM F2e females were reversed in HF+DM F3e. Additionally, HF+DM F3e females had an enrichment of additional damage/stress response pathways that were not previously seen in combined sexes (stress-activated protein kinase signaling cascade, ubiquitin-dependent protein catabolic process). Others have reported that female embryos display a more robust antioxidant capacity compared to males [118,119] and that sex-specific differences in DNA methylation and chromatin remodeling may contribute to sexual dimorphism in development and disease susceptibility [120]. Our study had limitations that should be considered when interpreting this data. Specifically, sex-specific genes may or may not be expressed at the E4.5 blastocyst stage. Our sub-set analyses included all non-male cells which includes females and indeterminate sexes (those not expressing Y chromosome genes). Despite this caveat, our findings add evidence that like ICM, female embryos may have inherent protective mechanisms that mitigate cellular stress and developmental programming across the female germline.

Mechanisms of developmental programming include DNA methylation, histone modification, chromatin remodeling, and non-coding RNAs [16,121], all of which exist on a spectrum of transient or permanent stability. DNA methylation is relatively more permanent because it can be maintained through mitosis, for example, in imprinting or X inactivation. While most histone modifications are reset during primordial germ cell development in mammals, preimplantation embryos can retain maternally inherited methylation sites and histone modifications including H3K27me3 repressive marks that confer imprinting, especially in TE [122,123]. Transcriptional changes due to chromatin remodeling and non-coding RNAs can be quickly reversed. Although this study did not directly examine epigenetic mechanisms, our pathway analyses and patterns of expression across generations inform potential mechanisms. For instance, we found that obese and diabetic pregnancy enriched chromatin-remodeling pathways in HF+DM F2e, but these pathways were not enriched in HF+DM F3e. Rather, HF+DM F3e had enriched pathways associated with RNA splicing. Although not a traditional form of epigenetic regulation, RNA splicing can also influence translation to incite a developmental change. Within the top 20 DEGs in HF+DM exposed embryos, several are known to be epigenetically regulated. For example, Id2 was downregulated in HF+DM F2e. There is evidence that Id2 expression is repressed by DNA methylation and that hypermethylation leads to premature stem cell maturation, a phenotype found in HF+DM F2e [95,96]. Zbtb46 was robustly upregulated in HF+DM F2e. Zbtb46 facilitates histone deacetylation, causing transcriptional silencing under the influence of inflammatory pathways [124]. Non-coding RNAs, like miRNAs, also repress gene expression by promoting mRNA degradation. Several DEGs that were downregulated in HF+DM F2e are reportedly repressed by miRNA (Ppp2ca, Ralb, Plcd1, Rasgrp2, Abcf3, and Naa20) [125,126,127,128,129,130]. Additionally, HF+DM F2e TE had pathway enrichment of small nuclear RNA (snRNA) processing, another type of non-coding RNA that is responsible for mRNA export and alternative splicing, additional reversible modifiers of expression. We identified a curious transcriptional pattern of Igfbp6 expression. Igfbp6 was significantly and robustly upregulated in HF+DM F2e. Although this upregulation persisted across generations, it was actually compounded in HF+DM F3e females. Igfbp6 has been reported to preferentially bind to and inhibit growth factor Igf-2 [77], which plays a critical role in embryonic response to oxidative stress [131]. The compounding generational pattern specific to females may again highlight that females have a more robust adaptation to oxidative stress.

Regardless of the underlying programming mechanism, our study confirms that the adverse in utero milieu of obese and diabetic pregnancy incites novel transcriptional changes during fetal primordial germ cell development to effect development of the grand-offspring. By carefully controlling confounding postnatal variables in our model, we demonstrated that the third trimester is a critical window of exposure that may be amenable to interventions. Technical innovation in our study included novel methods to grade preimplantation rat embryos and an innovative approach to scRNA-seq that overcomes barriers related to minimum cell requirements for the 10× Genomics platform. We incorporated a standardized number of HEK-293T cells with fresh blastomeres, then created libraries to process human and rat cell data separately. This allowed us to use fresh preimplantation embryos without the need for superovulation and cryopreservation which can disrupt mitochondria and confound transcriptomic data. We also analyzed transcriptomic variation across generations and did sub-set analyses by cell type and sex to delineate pathways that are reversibly and persistently programmed.

## 5. Conclusions

Others have reported the role of maternal high-fat diet and obesity on embryo and placental transcriptomics in the context of developmentally programmed metabolic disease [132,133,134,135,136,137]. To our knowledge, this is the first study to report transgenerational consequences from the compounding effects of maternal overnutrition and late-gestation diabetes. Overall, we found transcriptomic modifications in HF+DM F2e associated with mitochondrial dysfunction, oxidative stress, and altered epigenetic pathways that resulted in an advanced aging embryonic phenotype. On the other hand, we found novel, adaptive transcriptomic changes in HF+DM F3e that increased mitochondria quantity and cellular repair mechanisms, resulting in a delayed maturation embryonic phenotype. Importantly, these data establish that transcriptomic consequences persist across three generations, even outside of additional environmental exposures.

## Figures and Tables

**Table 1:** Embryo grading criteria.

	Poor	Good	Great
**Expansion Score** **(EXS)**	1: No discernable blastocoel cavity 2: Blastocoel present but is less than half of the embryo volume	3: Blastocoel that fills more than half of the embryo, but not fully expanded; space between blastomeres and zona pellucida	4: Fully expanded with a blastocoel that is flush with the zona pellucida
**Trophectoderm Score (TS)**	C: TE layer not cohesive or unable to be differentiated from other blastomeres	B: TE forms a cohesive layer but individual blastomeres can still be appreciated	A: TE forms a thin, cohesive layer
**Inner Cell Mass Score (ICMS)**	C: No discernable ICM or unable to be localized to one side of the embryo	B: ICM localized to one side but individual blastomeres can still be appreciated instead of being compact	A: ICM is localized to one side and completely compact

**Table 2:** F0 phenotype comparisons.

Parameter	CON n = 15	HF+DM n = 10	*p*-Value
Maternal age (weeks)	14.48 ± 0.48	15.16 ± 0.27	0.0913
Maternal age range (weeks)	13.14–19.29	14.00–16.00	0.0913
Weight at baseline (g)	208.20 ± 2.25	173.30 ± 2.40	**<0.0001**
Weight at post diet (g)	230.00 ± 2.80	252.10 ± 2.29	**<0.0001**
Weight at GD14 (g)	278.10 ± 4.88	302.60 ± 2.83	**0.0008**
Weight at P1 (g)	248.10 ± 3.53	273.40 ± 5.28	**0.0004**
Weight gain post diet (g)	21.80 ± 1.46	78.80 ± 2.83	**<0.0001**
Weight gain post diet to GD14 (g)	46.82 ± 3.35	51.00 ± 2.12	0.3299
Weight gain GD14 to P1 (g)	−37.64 ± 9.96	−29.11 ± 4.17	0.475
Weight gain post diet to P1 (g)	18.36 ± 3.73	21.89 ± 4.54	0.5563
Glucose at baseline (mg/dL)	103.10 ± 2.77	99.50 ± 2.69	0.387
Glucose post diet (mg/dL)	97.47 ± 3.31	89.60 ± 1.31	0.0749
Glucose GD15-GD21 (mg/dL)	87.50 ± 1.87	357.00 ± 18.62	**<0.0001**
Glucose at P1 (mg/dL)	108.60 ± 4.05	516.10 ± 35.43	**<0.0001**
Triglycerides at baseline (mg/dL)	74.16 ± 4.49	91.96 ± 6.16	**0.0254**
Triglycerides post diet (mg/dL)	92.40 ± 6.96	130.70 ± 12.63	**0.0131**
Triglycerides at P1 (mg/dL)	31.06 ± 4.52	122.20 ± 12.72	**<0.0001**
Total placentations at P1	13.42 ± 1.21	14.88 ± 0.72	0.5039
Live offspring at P1	11.14 ± 0.97	12.50 ± 1.91	0.1211

Data are expressed as mean ± SEM. Significant differences with *p* ≤ 0.05 are bolded. CON = control group, HF+DM = high-fat and diabetes group, P = postnatal day, GD = gestational day. g = grams.

**Table 3:** F1 offspring phenotype comparisons.

Parameter	CON n = 30	HF+DM n = 24	*p*-Value
Birthweight (g)	5.95 ± 0.12	6.15 ± 0.48	0.218
Adult weight (g)	286.00 ± 3.27	293.60 ± 2.74	0.0943
Maternal age (weeks)	20.09 ± 0.34	24.60 ± 0.57	**<0.0001**
Maternal age range (weeks)	17.00–23.43	21.43–30.71	**<0.0001**
Ovary weight (mg)	52.02 ± 1.27	62.57 ± 1.45	**<0.0001**
Ovary–body weight (mg/g)	0.18 ± 0.01	0.22 ± 0.01	**<0.0001**
Breeding days	2.35 ± 0.33	1.94 ± 0.23	0.5521
Embryos/litter	8.00 ± 0.94	9.90 ± 0.83	0.158
Glucose at E4.5 (mg/dL)	92.52 ± 3.00	94.79 ± 3.26	0.6107
Triglycerides at E4.5 (mg/dL)	109.40 ± 5.19	120.00 ± 6.19	0.1942

Data are expressed as mean ± SEM. Significant differences with *p* ≤ 0.05 are bolded. CON = control group, HF+DM = high-fat and diabetes group, E = embryonic day, g = grams.

**Table 4:** F2 offspring phenotype comparisons.

Parameter	CON n = 14	HF+DM n = 16	*p*-Value
Birthweight (g)	6.77 ± 0.20	6.75 ± 0.07	0.9228
Adult weight (g)	289.20 ± 3.83	270.90 ± 4.72	**0.0062**
Maternal age (weeks)	16.64 ± 0.54	16.82 ± 0.89	0.4162
Maternal age range (weeks)	11.43–20.00	13.71–27.86	0.4162
Ovary weight (mg)	45.84 ± 1.23	52.06 ± 2.04	**0.0146**
Ovary–body weight (mg)	0.16 ± 0.00	0.19 ± 0.01	**0.0005**
Breeding days	2.67 ± 0.30	2.30 ± 0.30	0.4106
Embryos/litter	10.25 ± 0.91	8.60 ± 0.79	**0.0459**
Glucose at E4.5 (mg/dL)	88.67 ± 4.05	98.50 ± 2.92	**0.0533**
Triglycerides at E4.5 (mg/dL)	114.70 ± 7.61	91.67 ± 9.76	0.0747

Data are expressed as mean ± SEM. Significant differences with *p* ≤ 0.05 are bolded. CON = control group, HF+DM = high-fat and diabetes group, E = embryonic day, g = grams.

**Table 5:** Top 20 DEGs between CON F2e and HF+DM F2e.

Gene ID	*p*-Value	Log2_FC	Adjusted *p*-Value
Id2	2.23 × 10^−6^	−1.81609	0.027468
AABR07025818.1	1.16 × 10^−5^	2.050856	0.142563
Abcf3	2.5 × 10^−5^	−3.593	0.306855
Naa20	3.43 × 10^−5^	−1.58134	0.42136
Ints7	0.000103	−2.32354	1
Mrpl3	0.000343	−1.64547	1
Bub3	0.000544	−1.745	1
H3f3c	0.000553	−1.2829	1
ENSRNOG00000066033	0.000611	−2.36167	1
Ifitm2	0.000663	1.348301	1
Ppp2ca	0.0007	−1.18861	1
Ilf2	0.000741	−1.2681	1
Plcd1	0.000753	−2.92558	1
Zbtb46	0.000971	3.74685	1
Rasgrp2	0.000971	3.74685	1
ENSRNOG00000021395	0.001041	2.009885	1
Ralb	0.001146	−2.83811	1
Eif2d	0.001146	−2.83811	1
Pafah1b2	0.001146	−3.16004	1
Ciapin1	0.001485	−1.51994	1

**Table 6:** Top 20 DEGs between CON F3e and HF+DM F3e.

Gene ID	*p*-Value	Log2_FC	Adjusted *p*-Value
Igfbp6	3.65 × 10^−19^	4.5062336	4.49 × 10^−15^
ENSRNOG00000066117	4.89 × 10^−17^	2.2258475	6.01 × 10^−13^
Rpl39.4	6.60 × 10^−12^	1.0457567	8.12 × 10^−8^
ENSRNOG00000021395	2.04 × 10^−11^	4.2120505	2.51 × 10^−7^
Rpl35al1	7.40 × 10^−11^	1.5173244	9.10 × 10^−7^
Snrpg.1	1.94 × 10^−9^	1.0166251	2.39 × 10^−5^
Pllp	3.17 × 10^−9^	1.0429563	3.8998 × 10^−5^
S100a10	6.55 × 10^−8^	1.3552269	0.00080551
Actn4	1.52 × 10^−7^	−1.0014088	0.00187237
Gng5	4.654 × 10^−7^	−1.5479987	0.00572219
Ckmt1	6.238 × 10^−7^	−1.035877	0.00767044
Arpp19.1	1.635 × 10^−6^	1.9381278	0.02009886
Ccne1	3.284 × 10^−6^	1.4799837	0.04037549
Ankrd11	3.403 × 10^−6^	−1.3870625	0.04184274
Eef1e1	5.506 × 10^−6^	1.0792953	0.06770111
ENSRNOG00000066033	9.522 × 10^−6^	−2.1043181	0.11708198
Rbx1	4.511 × 10^−5^	2.2120505	0.55472283
Rspry1	6.991 × 10^−5^	−3.9172325	0.85964477
Mrpl22	8.062 × 10^−5^	1.1104102	0.9912706
Mtres1	0.0001019	1.320864	0.55238095

**Table 7:** Top 20 DEGs between HF+DM F2e and HF+DM F3e.

Gene ID	*p*-Value	Log2_FC	Adjusted *p*-Value
Arf4	2.56 × 10^−6^	1.713882	0.03149491
Rpl35al1	3.23 × 10^−6^	1.1983587	0.03976302
Thumpd3	4.19 × 10^−6^	1.6076826	0.05146818
S100a10	7.05 × 10^−6^	1.3335076	0.08665579
Rac1	7.21 × 10^−6^	1.3608454	0.0886293
Mt-nd3	1.03 × 10^−5^	1.5909069	0.12687993
ENSRNOG00000067072	1.09 × 10^−5^	2	0.13350203
Mt-nd6	3.36 × 10^−5^	2.2306129	0.41298922
Bub3	5.29 × 10^−5^	1.6737718	0.6501446
Polr1g	5.8 × 10^−5^	1.3081223	0.71298689
Arfrp1	9.56 × 10^−5^	1.4150375	1
Phlda2	0.000132	3.1926451	1
Klhdc10	0.000132	3.3081223	1
Rpl9	0.000135	−1.266787	1
Naa20	0.000181	1.3225254	1
Hspa14	0.000213	1.474416	1
Ccz1b	0.000238	1.2097186	1
Atp1b1	0.000313	1.6265416	1
Pfdn4	0.000318	1.0036113	1
MGC116121	0.00047	−2.714246	1

## Data Availability

The single cell RNA-sequencing data curated from this study will be available in the online repository: https://www.ncbi.nlm.nih.gov/geo/, accesed on 3 June 2025, accession number: GSE298842 following an embargo period of 3 years from the date of publication to allow for additional group analyses paired with offspring phenotyping and an intervention.

## Supplementary Materials

The following supporting information can be downloaded at: https://www.mdpi.com/article/10.3390/biomedicines13082019/s1, Supplementary File (Word document): Table S1: Caloric content and fat differences in diets; Figure S1: Generational trees for control and high fat with diabetes exposure from F0 to F3 generations; Table S2: Average dam age and embryo sample size used across groups and generations; Figure S2: UMAP plot of total E4.5 embryonic cells classified by trophectoderm or inner cell mass; Table S3: Top 20 DEGs in TE cells between CON F2e and HF+DM F2e; Table S4: Top 20 DEGs in TE cells between CON F3e and HF+DM F3e; Table S5: Top 20 DEGs in ICM between CON F2e and HF+DM F2e; Table S6: Top 20 DEGs in ICM between CON F3e and HF+DM F3e; Table S7: HF+DM F1 versus HF+DM F2 dam phenotype comparisons; Table S8: Top 20 DEGs in TE between HF+DM F2e and HF+DM F3e; Table S9: Top 20 genes in ICM between HF+DM F2e and HF+DM F3e; Table S10: Top 20 DEGs in female/indeterminate cells between CON F2e and HF+DM F2e; Table S11: Top 20 DEGs in female/indeterminate cells between CON F3e and HF+DM F3e; Table S12: Top 20 DEGs in female/indeterminate cells between HF+DM F2e and HF+DM F3e; Figure S3: Exposure-mediated and generational transcriptomic analysis of E4.5 embryos in female/indeterminate cells; Figure S4: Group and generational base excision repair E4.5 F2 and F3 embryos. Table S13: Gene ID and corresponding name for genes used in manuscript. Supplementary File S2 Excel: Comparison of pathway analyses across groups and generations in total cells versus female/indeterminate cells.

## Abbreviations

The following abbreviations are used in this manuscript:

CON	Control
HF+DM	High fat+diabetes
F2e	Second-generation embryos
F3e	Third-generation embryos
STZ	Streptozotocin
E	Embryonic day
GD	Gestational day
P	Postnatal day
ROS	Reactive oxygen species
ETC	Electron transport chain
