# Supplementary material for: The Transgenerational Impact of High-Fat Diet and Diabetic Pregnancy on Embryonic Transcriptomics and Mitochondrial Health
Source: Biomedicines. 2025 Aug 19;13(8):2019. doi: 10.3390/biomedicines13082019 (PMC12383359; doi:10.3390/biomedicines13082019)
Supplement: Supplementary file 1 [file biomedicines-13-02019-s001.zip › Supplementary File.pdf]

Supplementary File

Table S1. Caloric content and fat differences of diets.

Diet	Caloric content (kcal/g)	Fat (% kcal)	Protein (% kcal)	CHO (% kcal)	Fat source
CD	3.2	16.6	22.9	60.5	Soybean oil
HF	4.3	39.7	18.8	41.4	Soybean oil, Primex (HVO)

Diet	Saturated (% daily intake)	MUFA (% daily intake)	PUFA (% daily intake)	Trans (% daily intake)	n6:n3 (% daily intake)
CD	3	4	11	0	9.6
HF	10.4	12	3.3	12.3	9.6

Diet	Palmitic 16:0 (% daily intake)	Stearic 18:0 (% daily intake)	Oleic 18:1 (% daily intake)	LA 18:2 n6 (% daily intake)	ALA 18:3 n3 (% daily intake)	ARA 20:4 n6 (% daily intake)
CD	0.7	0.2	1.2	3.1	0.3	0
HF	12.9	12.5	21.5	8.2	0.2	0.4

Caloric content and fatty acid levels of control diet, CD (TD.170868, Envigo Teklad Diets, Madison, WI) and high fat diet, HF (TD.95217, Envigo Teklad Diets, Madison, WI). Kcal: kilocalories; CHO: carbohydrates; HVO: hydrogenated vegetable oil; MUFA: monounsaturated fatty acids; PUFA: polyunsaturated fatty acids; LA: linoleic; ALA: alpha-linolenic; ARA: arachidonic.

Figure S1. Generational trees for control and high fat with diabetes exposure from F0 to F3 generations.

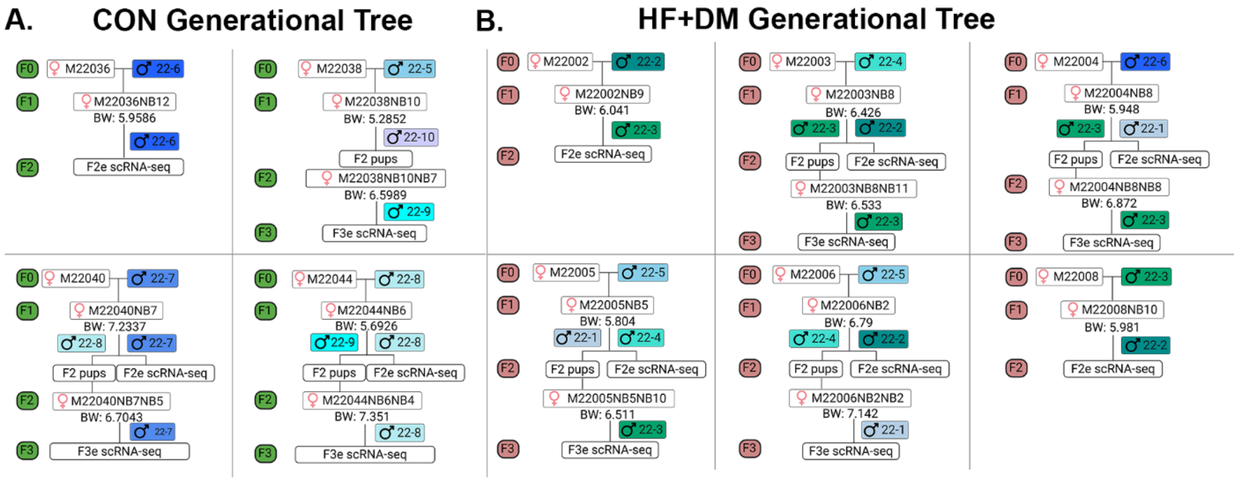

Figure S1. Generational trees for control and high fat with diabetes exposure from F0 to F3 generations. A breeding strategy was used to select a group of similar, control diet-fed sire breeders across generations. Each color of box represents an individual sire breeder used. Light gray squares represent the lineage from one F0 dam through F2/F3 generation embryos. F1 and F2 dams have their birth weight (BW) listed in grams below their name. Only animals used for single-cell RNA sequencing (scRNA-seq) experiments are listed. F2e = F2 generation E4.5 embryos. F3e = F3 generation E4.5 embryos. CON: controls, HF+DM: high fat + diabetes.

Table S2. Average dam age and embryo sample size used across groups and generations.

	CON F2e	HF+DM F2e	CON F3e	HF+DM F3e
Embryo grading (n)	229	189	121	125
Embryo staining (n)	25	26	11	12
Embryo scRNA-seq (n)	31	69	25	38

Figure S2. UMAP plot of total E4.5 embryonic cells classified by trophectoderm or inner cell mass.

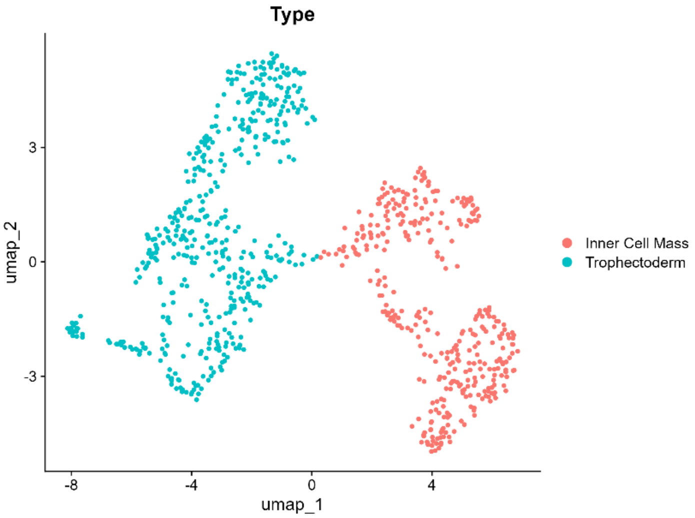

Figure S2. UMAP plot of total E4.5 embryonic cells classified by trophectoderm or inner cell mass. Cell population from single cell-RNA sequencing data from E4.5 embryos was separated into trophectoderm (TE) or inner cell mass (ICM) cells using pre-defined gene markers.

Table S3. Top 20 DEGs in TE cells between CON F2e and HF+DM F2e.

Gene ID	P-value	Log2_FC	Adjusted p-value
ENSRNOG00000028747	1.29847E-09	-1.151037757	1.59659E-05
Ddt	6.78832E-08	1.016601967	0.000834692
Dab2	4.66037E-07	1.075101455	0.005730387
ENSRNOG00000067072	4.62244E-06	-4.231325546	0.056837571
Snrpe	7.07498E-06	-1.022630974	0.086993956
H3f3c	1.44677E-05	-2.646363045	0.177894928
Rps4x	4.46717E-05	1.47701937	0.54928268
Naa20	4.88855E-05	-1.73947245	0.601096047
Vgll4	6.5802E-05	1.392317423	0.809101618
Id2	0.000133125	-1.499521657	1
ENSRNOG00000021395	0.000135841	2.587692293	1
Lamtor1	0.000159055	-3.031026896	1
Ppial4d	0.000236557	-1.628441137	1
ENSRNOG00000062325	0.00024005	-1.283792966	1
Ctbp1	0.000240908	-1.350907162	1
Snrpg	0.000346356	-1.107204234	1
Zranb2	0.000376782	1.257046981	1
Trappc10	0.000559775	1.480493234	1
RGD1311899	0.000929296	1.895530733	1

Rnf5	0.001002946	-2.02075856	1

Table S4. Top 20 DEGs in TE cells between CON F3e and HF+DM F3e.

Gene ID	P-value	Log2_FC	Adjusted p-value
ENSRNOG00000066117	4.55812E-08	2.538838451	0.000560467
Mt-atp6	1.1846E-06	1.097386954	0.014565833
Ccne1	3.05361E-06	1.558332661	0.037547169
Mt-nd2	3.32023E-06	1.297215458	0.040825523
S100a10	5.60535E-06	1.465223256	0.068923333
Cldn4	7.03463E-06	-1.076411813	0.086497861
Rpl35al1	8.26999E-06	2.207155104	0.10168776
Rpl39.4	8.44841E-06	1.07501761	0.103881605
ENSRNOG00000021395	1.16375E-05	3.880260756	0.143094732
Ndufb4	1.77546E-05	-1.207202086	0.218310799
Igfbp6	2.82875E-05	2.543225768	0.347823382
Actn4	3.2447E-05	-1.23062335	0.398967858
Lrrfip2	3.27418E-05	-2.312384322	0.402592958
Ckmt1	3.57562E-05	-1.060032998	0.439658263
Eef1e1	3.69024E-05	1.050185757	0.453751737
Snrpg.1	4.71346E-05	1.013743761	0.579567409
Slc7a6	4.72821E-05	-1.720131786	0.581380216
Psmg1	6.8981E-05	1.064685327	0.848190563
Men1	7.52452E-05	-4.119739244	0.925214864
Arpp19.1	0.000114739	2.202188851	1

Table S5. Top 20 DEGs in ICM between CON F2e and HF+DM F2e.

Gene ID	P-value	Log2_FC	Adjusted p-value
Aspm	0.00025413	3.527477006	1
Abcf3	0.00060653	-3.101879614	1
Cfap410	0.000712078	3.527477006	1
Ckb	0.001089218	-1.474325237	1
Zfpl1	0.001959277	3.164906927	1
Smpd1	0.00262464	-2.812372997	1
Cbx3	0.002658905	-1.864840417	1
Ubc	0.003085466	2.141823314	1
Bub3	0.003413663	-2.890375509	1
AABR07071244.1	0.004371948	2.135159583	1
Ttc7b	0.005312037	2.942514505	1
Rnf6	0.005312037	2.942514505	1
Tmem60	0.005312037	2.942514505	1
Slc16a1	0.005373302	-3.729910837	1
Plpp5	0.005373302	-2.812372997	1
Dimt1	0.005373302	-2.642447995	1
Ifitm2	0.006457393	1.500509958	1
Ptma.1	0.008638697	2.357552005	1
Mt-nd6	0.008813087	-1.449802917	1

Rab11a	0.009495216	-2.286304185	1

Table S6. Top 20 DEGs in ICM between CON F3e and HF+DM F3e.

Gene ID	P-value	Log2_FC	Adjusted p-value
Igfbp6	8.75052E-17	6.876453655	1.076E-12
ENSRNOG00000066117	4.8854E-11	1.86265946	6.0071E-07
ENSRNOG00000051885	1.54187E-10	0.74076431	1.8959E-06
Pa2g4	1.08999E-07	1.014178796	0.00134025
Actn4	2.39185E-06	-1.185766611	0.02941023
ENSRNOG00000021395	9.86537E-06	3.749921249	0.12130454
Pdcd11	1.04683E-05	-1.981397782	0.12871861
Rpl35al1	1.68786E-05	1.122184725	0.20753916
Arfrp1	4.06287E-05	1.38735117	0.49957088
Lamtor1	4.46169E-05	-1.519539426	0.54860886
ENSRNOG00000066153	4.79804E-05	-1.510606301	0.58996665
Trappc4	4.81591E-05	2.186985055	0.59216466
Afg3l2	5.80568E-05	-3.857409064	0.71386662
Fbxo15	5.9759E-05	-1.572006846	0.73479684
Gng5	6.03931E-05	-1.604428323	0.74259363
Ppp1r10	8.03962E-05	-2.964324268	0.98855191
Haus8	0.000136241	-3.616400965	1
Zfpm1	0.000136241	-3.616400965	1
Becn1	0.000196962	-2.25650502	1
Dab2	0.00025572	-1.373102777	1

Table S7. HF+DM F1 versus HF+DM F2 dam phenotype comparisons.

Parameter	HF+DM F1 n=24	HF+DM F2 n=16	P-value
Birth weight (g)	6.39±0.16	6.75±0.07	**0.0443**
Adult weight (range) (g)	292.20±3.65	270.90±4.72	**0.0016**
Maternal age (weeks)	24.60±0.57	16.82±0.89	**<0.0001**
Maternal age range (weeks)	21.43-30.71	13.71-27.86	N/A
Ovary weight (mg)	62.57±1.45	52.06±2.04	**<0.0001**
Ovary:body weight (mg/g)	0.22±0.01	0.19±0.01	**0.0106**
Breeding days	1.94±0.23	2.30±0.30	0.4214
Embryos/litter	9.90±0.83	8.60±0.79	0.2059
Glucose at E4.5 (mg/dL)	94.79±3.26	98.50±2.92	0.4312
Triglycerides at E4.5 (mg/dL)	120.00±6.19	91.67±9.76	**0.0143**

Table S8. Top 20 DEGs in TE between HF+DM F2e and HF+DM F3e.

Gene ID	P-value	Log2_FC	Adjusted p-value
Snrpe	6.14731E-09	1.181868866	7.55873E-05
ENSRNOG00000067072	7.4E-09	4.51967104	9.09904E-05

Rpl39.4	6.33606E-08	1.268378488	0.000779081
Sec61g	8.3456E-08	1.017764279	0.001026175
Rac1	9.54208E-08	1.632680002	0.001173294
S100a10	2.02185E-07	1.663854629	0.002486069
Naa30	2.36413E-07	1.501749132	0.002906933
Rpa3	1.31731E-06	1.058966643	0.016197617
Mt-nd3	1.56812E-06	1.770935765	0.019281585
Rbis	2.12181E-06	1.349746039	0.026089744
Naa20	2.57037E-06	1.752710706	0.031605325
Ctbp1	3.58351E-06	1.267283879	0.044062853
Arf4	6.44101E-06	1.70820001	0.079198623
Ruvbl1	1.00365E-05	2.174174474	0.123408362
Bub3	1.80785E-05	1.166881982	0.222293768
Cpne8	3.64988E-05	-1.126963571	0.448789835
Map7	4.12524E-05	-1.054644216	0.507239599
Cdkn2aipnl	6.4129E-05	1.086711633	0.788530357
Snrpg.1	6.6072E-05	1.654995393	0.812421717
ENSRNOG00000070284	7.08398E-05	-1.346247774	0.871046128

Table S9. Top 20 DEGs in ICM between HF+DM F2e and HF+DM F3e.

Gene ID	P-value	Log2_FC	Adjusted p-value
Ftl1	5.05664E-06	-1.131294303	0.062176411
Gdi2	0.000245215	-1.168196602	1
Sox13	0.000255757	-4.184875343	1
ENSRNOG00000048258	0.00035894	1.543045112	1
AABR07071244.1	0.0007033	-1.541019153	1
Haus8	0.001251748	-4.407267764	1
Pdia6	0.001282806	2.622479579	1
Zfp326	0.001341377	2.722015253	1
Atp6ap1	0.001341377	2.923649114	1
Krtcap2	0.00147744	2.01899699	1
Timm50	0.0024044	-1.401973465	1
Prpf18	0.002500814	2.400087158	1
Ergic2	0.00338743	-1.160627797	1
ENSRNOG00000037610	0.003904044	2.487549999	1
Slc16a1	0.003904044	3.609540523	1
Txndc15	0.003904044	2.570012159	1
ENSRNOG00000056651	0.004252034	-1.262877855	1
Orc6	0.00449432	-1.184875343	1
Spc25	0.005549103	2.722015253	1
Dmtf1	0.005549103	2.400087158	1

Table S10. Top 20 DEGs in female/indeterminate cells between CON F2e and HF+DM F2e.

Gene ID	P-value	Log2_FC	Adjusted p-value
Mrpl3	0.000120935	-2.064742765	1

Ciapin1	0.000123988	1.963826387	1
Ifitm2	0.000205639	1.475232281	1
Rpl38.1	0.000259041	-1.099270448	1
Abcf3	0.000396006	-3.147204925	1
Ube2z	0.000632097	-3.225207437	1
Naa20	0.000710383	-1.649705265	1
Id2	0.000756457	-1.460797161	1
Bub1	0.000880768	1.022720077	1
Ttc4	0.001007176	-3.064742765	1
Borcs8	0.001007176	-2.977279923	1
Pom121	0.001338409	1.940257916	1
Uba2	0.001385087	-1.10656294	1
Ythdc1	0.001520224	1.101791647	1
H3f3c	0.001520863	-1.342929396	1
Sf3b4	0.001971757	-1.601770788	1
ENSRNOG00000067072	0.002305194	-1.954559847	1
RGD1306271	0.002325175	1.135194806	1
Csrp1	0.002483986	-2.270061673	1
ENSRNOG00000066033	0.002532259	-2.479780264	1

Table S11. Top 20 DEGs in female/indeterminate cells between CON F3e and HF+DM F3e.

Gene ID	P-value	Log2_FC	Adjusted p-value
Igfbp6	1.65082E-17	5.022367813	2.02985E-13
ENSRNOG00000066117	2.86989E-10	1.854190839	3.52881E-06
Rpl39.4	1.79837E-08	1.011972642	0.000221128
ENSRNOG00000021395	3.28198E-08	4.285402219	0.000403552
Rpl35al1	3.77065E-07	1.236376651	0.004636387
Actn4	2.85384E-06	-1.208586622	0.03509085
Ckmt1	1.36682E-05	-1.006952761	0.168064761
Arpp19.1	2.54334E-05	2.209453366	0.312728811
Gng5	4.90258E-05	-1.717600269	0.602820938
Ndufaf6	0.000127083	2.459431619	1
Mapk1ip1l	0.00014156	-2.152003093	1
Shcbp1	0.000160694	1.070389328	1
Mapk1	0.00025436	-1.473931188	1
Usp19	0.000271477	-1.112474729	1
Pde4d	0.000470307	-2.662965013	1
Eef1e1	0.000551463	1.057450272	1
Fbxo15	0.000678282	-1.30580843	1
Zcchc7	0.000739444	-2.103093493	1
Atp12a	0.000856252	-3.415037499	1
Ccne1	0.000867062	1.321928095	1

Table S12. Top 20 DEGs in female/indeterminate cells between HF+DM F2e and HF+DM F3e.

Gene ID	P-value	Log2_FC	Adjusted p-value
Polr1g	1.1475E-06	1.510961919	0.0141097

Hspa14	8.62605E-06	2.121015401	0.106065959
ENSRNOG00000067072	1.76488E-05	2.274261661	0.217009175
Phlda2	0.000117882	3.240314329	1
Arfrp1	0.000168134	1.746117293	1
Asxl1	0.000182867	3.371558863	1
Mta3	0.00018654	1.085036104	1
Mt-nd6	0.000230488	2.499048598	1
Ciapin1	0.000326188	2.503348735	1
Igfbp6	0.000388542	1.063009798	1
Naa20	0.000434713	1.548436625	1
Celf1	0.000583144	2.169925001	1
Thumpd3	0.000585166	1.307428525	1
Mt-nd3	0.000619059	1.592830744	1
Nras	0.000681468	2.935459748	1
Tmem59	0.000794644	1.402585758	1
Rac1	0.000928546	1.202839624	1
Commd2	0.000986886	1.005538184	1
Arf4	0.001002895	1.548436625	1
Atmin	0.001040242	2.847996907	1

Figure S3. Exposure-mediated and generational transcriptomic analysis of E4.5 embryos in female/indeterminate cells.

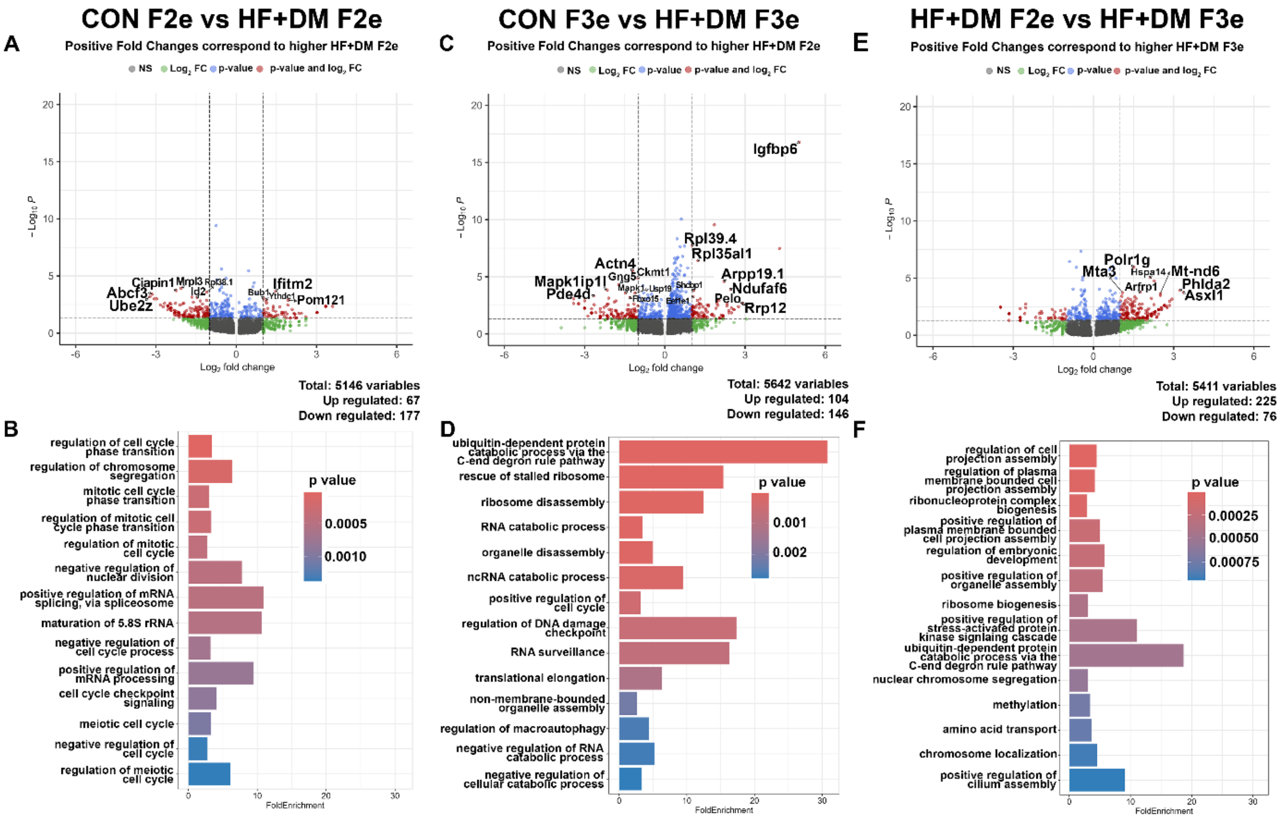

Figure S3. Exposure-mediated and generational transcriptomic analysis of E4.5 embryos in female/indeterminate cells. Differences between E4.5 embryos from CON F2e vs. HF+DM F2e (A,B), CON F3e vs. HF+DM F3e (C,D), and HF+DM F2e vs. HF+DM F3e (E,F) in "female/indeterminate" cells, in which cells expressing male markers (Eif2s3y, Usp9y, and/or Kmd5d) were removed from dataset. Volcano plots (A,C,E) represent all DEGs between groups with red dots representing significance. Significant DEGs were used for pathway analyses (B,D,F) by fold enrichment of genes sorted by p-value.

Figure S4. Group and generational base excision repair in E4.5 F2 and F3 embryos.

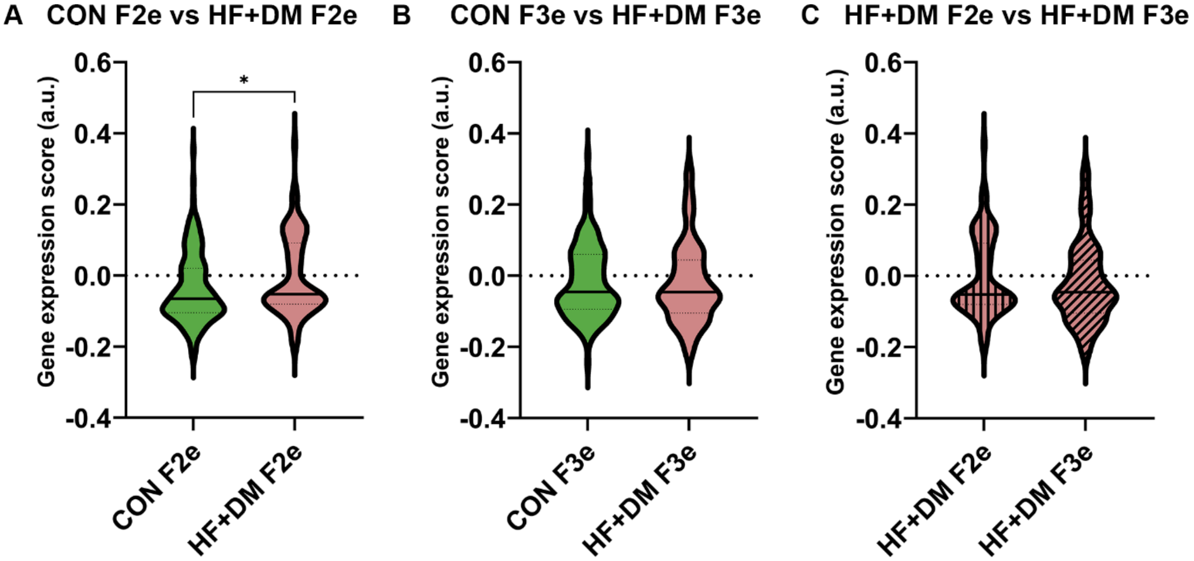

Figure S4. Group and generational base excision repair in E4.5 F2 and F3 embryos. Gene expression score using scRNA-seq data from E4.5 embryos was generated using genes involved in base excision repair (BER) mechanism. Differences are shown between CON F2e vs. HF+DM F2e (A), CON F3e vs. HF+DM F3e (B), and HF+DM F2e vs. HF+DM F3e (C). Violin plots represent median with quartiles. *p<0.05.

Table S13. Gene ID and corresponding name for genes used in manuscript.

Gene ID	Name
AABR07025818.1	N/A
Abcf3	ATP binding cassette subfamily F member 3
Abhd10	abhydrolase domain containing 10, depalmitoylase
Acadm	acyl-CoA dehydrogenase medium chain
Acads	acyl-CoA dehydrogenase short chain
Aco2	aconitase 2
Actn4	actinin alpha 4
Afg3l1	AFG3 (ATPase family gene 3)-like 1
Ahcyl1	adenosylhomocysteinase-like 1
Akap7	A-kinase anchoring protein 7
Aldh1b1	aldehyde dehydrogenase 1 family, member B1
Aldh4a1	aldehyde dehydrogenase 4 family, member A1
Aldh7a1	aldehyde dehydrogenase 7 family, member A1

Alkbh7	alkB homolog 7
Amt	aminomethyltransferase
Angel2	angel homolog 2
Ankrd11	ankyrin repeat domain containing 11
Arf4	ARF GTPase 4
Arfrp1	ARF related protein 1
Arpp19.1	cAMP-regulated phosphoprotein 19
Aspm	assembly factor for spindle microtubules
Asxl1	ASXL transcriptional regulator 1
Atad1	ATPase family, AAA domain containing 1
Atmin	ATM interactor
Atp12a	ATPase H+/K+ transporting non-gastric alpha2 subunit
Atp1b1	ATPase Na+/K+ transporting subunit beta 1
Bcs1l	BCS1 homolog, ubiquinol-cytochrome c reductase complex chaperone
Bdh1	3-hydroxybutyrate dehydrogenase 1
Becn1	beclin 1
Bhmt	betaine-homocysteine S-methyltransferase
Bin3	bridging integrator 3
Bloc1s1	biogenesis of lysosomal organelles complex-1, subunit 1
Bmpr2	bone morphogenetic protein receptor type 2
Bnip3l	BCL2 interacting protein 3 like
Borcs6	BLOC-1 related complex subunit 6
Borcs8	BLOC-1 related complex subunit 8
Bub1	BUB1 mitotic checkpoint serine/threonine kinase
Bub3	BUB3 mitotic checkpoint protein
Cables2	Cdk5 and Abl enzyme substrate 2
Cbx3	chromobox 3
Ccdc127	coiled-coil domain containing 127
Ccdc17	coiled-coil domain containing 17
Ccna2	cyclin A2
Ccne1	cyclin E1
Ccz1b	CCZ1 homolog, vacuolar protein trafficking and biogenesis associated
Cd63	Cd63 molecule
Celf1	CUGBP, Elva-like family member 1
Cep89	centrosomal protein 89
Cfap410	cilia and flagella associated protein 410
Chchd2	coiled-coil-helix-coiled-coil-helix domain containing 2
Ciapin1	cytokine induced apoptosis inhibitor 1
Ckb	creatine kinase B
Ckmt1	creatine kinase, mitochondrial 1
Cldn4	claudin 4
Cog6	component of oligomeric golgi complex 6
Commd2	COMM domain containing 2

Coq6	coenzyme Q6 monooxygenase
Coq9	coenzyme Q9
Cox19	cytochrome c oxidase assembly factor COX19
Cpne8	copine 8
Csrp1	cysteine and glycine-rich protein 1
Ctbp1	C-terminal binding protein 1
Cyb5r3	cytochrome b5 reductase 3
Dab2	DAB adaptor protein 2
Ddt	D-dopachrome tautomerase
Dera	deoxyribose-phosphate aldolase
Dhrs7b	dehydrogenase/reductase 7B
Diablo	diablo, IAP-binding mitochondrial protein
Dld	dihydrolipoamide dehydrogenase
Dlst	dihydrolipoamide S-succinyltransferase
Dnajc4	DnaJ heat shock protein family (Hsp40) member C4
Dnm1l	dynamin 1-like
Dnttip2	deoxynucleotidyltransferase, terminal, interacting protein 2
Ebp	EBP, cholestenol delta-isomerase
Eef1e1	eukaryotic translation elongation factor 1 epsilon 1
Eif2d	eukaryotic translation initiation factor 2D
ENSRNOG00000004262	nuclear ubiquitous casein and cyclin-dependent kinase substrate 1-like
ENSRNOG00000021395	ribosomal protein S8, pseudogene 17
ENSRNOG00000066033	MARCKS-related protein-like
ENSRNOG00000066117	ribosomal protein L37A, pseudogene 1
ENSRNOG00000067072	Hmgb1l2, high mobility group box 1 like 2
Ervfrd-1	endogenous retrovirus group FRD member 1, envelope
Ethe1	ETHE1, persulfide dioxygenase
Fam177a1	family with sequence similarity 177, member A1
Fam83h	family with sequence similarity 83, member H
Fastkd3	FAST kinase domains 3
Fbxo15	FAST kinase domains 3
Fdx2	ferredoxin 2
Fdxr	ferredoxin reductase
Fgd4	FYVE, RhoGEF and PH domain containing 4
Fis1	fission, mitochondrial 1
Fkbp2	FKBP prolyl isomerase 2
Flad1	flavin adenine dinucleotide synthetase 1
Fnta	farnesyltransferase, CAAX box, subunit alpha
Ftl1	ferritin light chain 1
Fundc1	FUN14 domain containing 1
Gatc	glutamyl-tRNA amidotransferase subunit C
Gdi2	GDP dissociation inhibitor 2
Gfus	GDP-L-fucose synthase

Gldc	glycine decarboxylase
Glrx2	glutaredoxin 2
Gng5	G protein subunit gamma 5
Gpx1	glutathione peroxidase 1
Gpx2	glutathione peroxidase 2
Grhpr	glyoxylate and hydroxypyruvate reductase
Gsr	glutathione-disulfide reductase
Gtpbp10	GTP binding protein 10
H3f3c	H3 histone, family 3C
Hadhb	hydroxyacyl-CoA dehydrogenase trifunctional multienzyme complex subunit beta
Haus8	HAUS augmin-like complex, subunit 8
Hccs	holocytochrome c synthase
Hdhd2	haloacid dehalogenase-like hydrolase domain containing 2
Hibadh	3-hydroxyisobutyrate dehydrogenase
Hibch	3-hydroxyisobutyryl-CoA hydrolase
Homer3	homer scaffold protein 3
Hspa14	heat shock protein family A (Hsp70) member 14
Htatip2	HIV-1 Tat interactive protein 2
Hyal3	hyaluronidase 3
Id2	inhibitor of DNA binding 2
Idh2	isocitrate dehydrogenase (NADP(+)) 2
Ifitm2	interferon induced transmembrane protein 2
Ilf2	interleukin enhancer binding factor 2
Ints7	integrator complex subunit 7
Isg20l2	interferon stimulated exonuclease gene 20-like 2
Kcnc4	potassium voltage-gated channel subfamily C member 4
Klhdc10	kelch domain containing 10
Lactb	lactamase, beta
Lamtor1	late endosomal/lysosomal adaptor, MAPK and MTOR activator 1
Ldhal6b	lactate dehydrogenase A-like 6B
Letm1	leucine zipper and EF-hand containing transmembrane protein 1
Lrpprc	leucine-rich pentatricopeptide repeat containing
Lrrfip2	LRR binding FLII interacting protein 2
Lyrm9	LYR motif containing 9
Map7	microtubule-associated protein 7
Map3k7	mitogen activated protein kinase kinase kinase 7
Mapk1	mitogen activated protein kinase 1
Mapklip1l	mitogen activated protein kinase 1 interacting protein 1 like
Mccc2	methylcrotonyl-CoA carboxylase subunit 2
Men1	menin 1
MGC116121	similar to human chromosome 18 open reading frame 21
Micos13	mitochondrial contact site and cristae organizing system subunit 13
Micu1	mitochondrial calcium uptake 1

Micu2	mitochondrial calcium uptake 2
Mief1	mitochondrial elongation factor 1
Mpg	N-methylpurine-DNA glycosylase
Mpv17	mitochondrial inner membrane protein MPV17
Mrm3	mitochondrial rRNA methyltransferase 3
Mrpl2	mitochondrial ribosomal protein L2
Mrpl20	mitochondrial ribosomal protein L20
Mrpl22	mitochondrial ribosomal protein L22
Mrpl3	mitochondrial ribosomal protein L3
Mrpl39	mitochondrial ribosomal protein L39
Mrpl42	mitochondrial ribosomal protein L42
Mrpl9	mitochondrial ribosomal protein L9
Mrps23	mitochondrial ribosomal protein S23
Mrps34	mitochondrial ribosomal protein S34
Mrps36	mitochondrial ribosomal protein S36 like 1
Mta3	metastasis associated 1 family, member 3
Mt-atp6	ATP synthase subunit a
Mt-atp8	ATP synthase F(0) complex subunit 8
Mtch2	mitochondrial carrier 2
Mt-co1	mitochondrially encoded cytochrome c oxidase I
Mt-co2	mitochondrially encoded cytochrome c oxidase II
Mt-co3	mitochondrially encoded cytochrome c oxidase III
Mt-cyb	mitochondrially encoded cytochrome b
Mtfmt	mitochondrial methionyl-tRNA formyltransferase
Mtg1	mitochondrial ribosome-associated GTPase 1
Mt-nd2	mitochondrially encoded NADH:ubiquinone oxidoreductase core subunit 2
Mt-nd3	mitochondrially encoded NADH:ubiquinone oxidoreductase core subunit 3
Mt-nd4	mitochondrially encoded NADH:ubiquinone oxidoreductase core subunit 4
Mt-nd4l	mitochondrially encoded NADH:ubiquinone oxidoreductase core subunit 4L
Mt-nd5	mitochondrially encoded NADH:ubiquinone oxidoreductase core subunit 5
Mt-nd6	mitochondrially encoded NADH:ubiquinone oxidoreductase core subunit 6
Mtres1	mitochondrial transcription rescue factor 1
Mtx2	metaxin 2
Mutyh	mutY DNA glycosylase
Naa20	N(alpha)-acetyltransferase 20, NatB catalytic subunit
Naa30	N(alpha)-acetyltransferase 30, NatC catalytic subunit
Ndufa8	NADH:ubiquinone oxidoreductase subunit A8
Ndufa9	NADH:ubiquinone oxidoreductase subunit A9
Ndufaf6	NADH:ubiquinone oxidoreductase complex assembly factor 6
Ndufb4	NADH:ubiquinone oxidoreductase subunit B4
Ndufs5	NADH:ubiquinone oxidoreductase subunit S5
Npc1	NPC intracellular cholesterol transporter 1
Nras	NRAS proto-oncogene, GTPase

Nthl1	nth-like DNA glycosylase 1
Obp2a	odorant binding protein 2A
Ociad1	OCIA domain containing 1
Ogg1	8-oxoguanine DNA glycosylase
Pa2g4	proliferation-associated 2G4
Pafah1b2	platelet-activating factor acetylhydrolase 1b, catalytic subunit 2
Pan3	poly(A) specific ribonuclease subunit PAN3
Pars2	prolyl-tRNA synthetase 2, mitochondrial
Pccb	propionyl-CoA carboxylase subunit beta
Pde4d	phosphodiesterase 4D
Pdhb	pyruvate dehydrogenase E1 subunit beta
Pdk3	pyruvate dehydrogenase kinase 3
Pfdn4	prefoldin subunit 4
Pgs1	phosphatidylglycerophosphate synthase 1
Phlda2	pleckstrin homology-like domain, family A, member 2
Pithd1	PITH domain containing 1
Plcd1	phospholipase C, delta 1
Plgrkt	plasminogen receptor with a C-terminal lysine
Pllp	plasmolipin
Pmaip1	phorbol-12-myristate-13-acetate-induced protein 1
Pmpca	peptidase, mitochondrial processing subunit alpha
Pnpo	pyridoxamine 5'-phosphate oxidase
Polb	DNA polymerase beta
Polg2	DNA polymerase gamma 2, accessory subunit
Polr1g	RNA polymerase I subunit G
Pom121	POM121 transmembrane nucleoporin
Ppial4d	peptidylprolyl isomerase A (cyclophilin A)-like 4D
Ppox	protoporphyrinogen oxidase
Ppp1r10	protein phosphatase 1, regulatory subunit 10
Ppp2ca	protein phosphatase 2 catalytic subunit alpha
Prdx1	peroxiredoxin 1
Prdx2	peroxiredoxin 2
Prdx3	peroxiredoxin 3
Prdx5	peroxiredoxin 5
Prdx6	peroxiredoxin 6
Psmg1	proteasome assembly chaperone 1
Ptges2	prostaglandin E synthase 2
Qdpr	quinoid dihydropteridine reductase
Rac1	Rac family small GTPase 1
Ralb	RAS like proto-oncogene B
Rasgrp2	RAS guanyl releasing protein 2
Rbis	ribosomal biogenesis factor
Rbx1	ring-box 1

Rexo2	RNA exonuclease 2
RGD1306271	KIAA1549 homolog (Kiaa1549)
Rhbdd2	rhomboid domain containing 2
Rhot2	ras homolog family member T2
Rpa3	replication protein A3
Rpia	ribose 5-phosphate isomerase A
Rpl35al1	ribosomal protein L35A like 11
Rpl38.1	Ribosomal protein L38
Rpl39	ribosomal protein L39
Rpl39.4	ribosomal protein L39
Rpl9	ribosomal protein L9
Rspry1	ring finger and SPRY domain containing 1
Rtn4ip1	reticulon 4 interacting protein 1
Ruvbl1	RuvB-like AAA ATPase 1
S100a10	S100 calcium binding protein A10
Sdhaf2	succinate dehydrogenase complex assembly factor 2
Sdhaf3	succinate dehydrogenase complex assembly factor 3
Sec61g	Sec61 translocon subunit gamma
Septin4	septin 4
Serpinb9	serpin family B member 9
Sf3b4	splicing factor 3B subunit 4
Shcbp1	SHC binding and spindle associated 1
Slc16a1	solute carrier family 16 member 1
Slc25a10	solute carrier family 25 member 10
Slc25a20	solute carrier family 25 member 20
Slc25a25	solute carrier family 25 member 25
Slc25a44	solute carrier family 25, member 44
Slirp	SRA stem-loop interacting RNA binding protein
Smagp	small cell adhesion glycoprotein
Snap29	synaptosome associated protein 29
Snd1	staphylococcal nuclease and tudor domain containing 1
Snrpe	small nuclear ribonucleoprotein polypeptide E
Snrpg	small nuclear ribonucleoprotein polypeptide G
Snrpg.1	small nuclear ribonucleoprotein polypeptide G
Sod1	superoxide dismutase 1
Sod2	superoxide dismutase 2
Sox13	SRY-box transcription factor 13
Sycp3	synaptonemal complex protein 3
Tceal8	transcription elongation factor A like 8
Ttc4	tetratricopeptide repeat domain 4
Tct19	tetratricopeptide repeat domain 19
Tdg	thymine-DNA glycosylase
Them4	thioesterase superfamily member 4

Thumpd3	THUMP domain containing 3
Timm22	translocase of inner mitochondrial membrane 22
Timm50	translocase of inner mitochondrial membrane 50
Timm8a2	translocase of inner mitochondrial membrane 8A2
Tmed1	transmembrane p24 trafficking protein 1
Tmem126b	transmembrane protein 126B
Tmem175	transmembrane protein 175
Tmem238	transmembrane protein 238
Tmem59	Transmembrane protein 59
Trappc4	trafficking protein particle complex subunit 4
Trit1	tRNA isopentenyltransferase 1
Trub1	TruB pseudouridine synthase family member 1
Trub2	TruB pseudouridine synthase family member 2
Twnk	twinkle mtDNA helicase
Txnrd1	thioredoxin reductase 1
Txnrd2	thioredoxin reductase 2
Uba2	ubiquitin-like modifier activating enzyme 2
Ubc	ubiquitin C
Ube2z	ubiquitin-conjugating enzyme E2Z
Ung	uracil-DNA glycosylase
Uqcc3	ubiquinol-cytochrome c reductase complex assembly factor 3
Uqcrfs1	ubiquinol-cytochrome c reductase, Rieske iron-sulfur polypeptide 1
Usp19	ubiquitin specific peptidase 19
Vgll4	vestigial-like family member 4
Ythdc1	YTH N6-methyladenosine RNA binding protein C1
Zcchc7	zinc finger CCHC-type containing 7
Zbtb46	zinc finger and BTB domain containing 46
Zfp958	zinc finger protein 958
Zfpl1	zinc finger protein-like 1
Zfpm1	zinc finger protein, multitype 1
Zfyve21	zinc finger FYVE-type containing 21
Zranb2	zinc finger RANBP2-type containing 2
